# Association Between the COVID-19 Pandemic and Early Childhood Development

**DOI:** 10.1001/jamapediatrics.2023.2096

**Published:** 2023-07-10

**Authors:** Koryu Sato, Taiyo Fukai, Keiko K. Fujisawa, Makiko Nakamuro

**Affiliations:** 1 Department of Social Epidemiology, Graduate School of Medicine and School of Public Health, Kyoto University, Kyoto, Japan; 2 Graduate School of Media and Governance, Keio University, Kanagawa, Japan; 3 Institute of Humanities and Social Sciences, University of Tsukuba, Ibaraki, Japan; 4 The Tokyo Foundation for Policy Research, Tokyo, Japan; 5 Department of Education, Faculty of Letters, Keio University, Tokyo, Japan; 6 Faculty of Policy Management, Keio University, Kanagawa, Japan

## Abstract

**Importance:**

Although a growing number of studies have reported negative associations of the COVID-19 pandemic with academic performance among school-aged children, less is known about the pandemic’s association with early childhood development.

**Objective:**

To examine the association between the COVID-19 pandemic and early childhood development.

**Design, Setting, and Participants:**

In this cohort study conducted in all accredited nursery centers in a Japanese municipality, baseline surveys of children aged 1 and 3 years (1000 and 922, respectively) were conducted between 2017 and 2019, and participants were followed up for 2 years.

**Exposure:**

Children’s development was compared at age 3 or 5 years between cohorts that were exposed to the pandemic during the follow-up and a cohort that was not.

**Main Outcome and Measure:**

Children’s developmental age was measured by nursery teachers using the Kinder Infant Development Scale (KIDS). Data were analyzed between December 8, 2022, and May 6, 2023.

**Results:**

A total of 447 children (201 girls [45.0%] and 246 boys [55.0%]) aged 1 year at baseline were followed up to age 3 years, and 440 children (200 girls [45.5%] and 240 boys [54.5%]) aged 3 years at baseline were followed up to age 5 years. During the follow-up, the cohorts that were exposed to the pandemic were 4.39 months behind in development at age 5 compared with the cohort that was not (coefficient, −4.39; 95% credible interval, −7.66 to −1.27). Such a negative association was not observed in development at age 3 years (coefficient, 1.32; 95% credible interval, −0.44 to 3.01). Variations in development were greater during the pandemic than before the pandemic regardless of age. Additionally, the quality of care at nursery centers was positively associated with development at age 3 years during the pandemic (coefficient, 2.01; 95% credible interval, 0.58-3.44), while parental depression appeared to amplify the association between the pandemic and delayed development at age 5 (coefficient of interaction, −2.62; 95% credible interval, −4.80 to −0.49; *P* = .009).

**Conclusions and Relevance:**

The findings of this study showed an association between exposure to the pandemic and delayed childhood development at age 5 years. Variations in development widened during the pandemic regardless of age. It is important to identify children with developmental delays associated with the pandemic and provide them with support for learning, socialization, physical and mental health, and family support.

## Key Points

**Question:**

How was the COVID-19 pandemic associated with early childhood development?

**Findings:**

This cohort study in 447 children aged 1 to 3 years and 440 children aged 3 to 5 years in Japan found that children exposed to the pandemic were 4.39 months behind in development at age 5 years compared with those not exposed to the pandemic. Variations in development were greater during the pandemic vs the prepandemic period regardless of age.

**Meaning:**

These findings suggest the importance of identifying children with delayed development associated with the pandemic and providing them with support for learning, socialization, physical and mental health, and family support.

## Teaser

This cohort study examined the association between COVID-19 and early childhood development in Japanese children.

## Introduction

The COVID-19 pandemic has affected children’s lives. Bronfenbrenner’s ecologic systems theory identifies 5 multilayered systems that are pivotal in child development: microsystem (eg, family and nursery), mesosystem (eg, a parent-teacher relationship), exosystem (eg, neighbors and parental workplace), macrosystem (eg, culture and economy), and chronosystem (eg, historical circumstances).^1,2^ The pandemic disturbed all of these systems. Studies have shown evidence of increased mental health issues,^3,4^ impaired sleep quality,^3^ decreased physical activity,^5,6,7^ weight gain,^8,9^ and increased screen time^3,5^ among children during the pandemic. A systematic review and meta-analysis reported that the pandemic was negatively associated with the academic performance of school-aged children, especially among primary school students and children from low socioeconomic backgrounds.^10,11^ However, it is still unclear how the pandemic might have affected early childhood development.

Few studies have explored the association between the pandemic and development among infants and preschoolers. An Italian cross-sectional, online-based study observed increased emotional symptoms and self-regulation difficulties during the quarantine period among children aged 2 to 5 years in reports from 245 mothers.^12^ An ecologic study at 3 preschools in Japan involving 32 children aged 4 to 5 years examined their socioemotional skills as assessed by teachers and reported that children in a preschool that had a school recital during the pandemic presented higher socioemotional skills than those whose schools canceled recitals.^13^ However, these studies have several limitations, including the use of convenience sampling, small sample sizes, and possible bias because of the unavailability of prepandemic and individual data. Thus, a cohort study using a rigorous sampling method and individual data is required to obtain more precise insights. Moreover, the pandemic disproportionately affected children with low socioeconomic status and parents with depression, which could widen disparities in child development.^10,11,14^ Nonetheless, most studies did not explore how variations in development altered during the pandemic, reporting instead the mean change in the population. To fill the existing knowledge gaps, this study examined the association between the COVID-19 pandemic and early childhood development by using census-based cohort data from a Japanese municipality, investigating not only the population mean but also variations in development.

## Methods

### Setting and Participants

In this cohort study, we studied children aged 1, 3, and 5 years as of April 1 of each year who attended accredited nursery centers in a municipality located in the suburbs of Tokyo, Japan. In Japan, 98.3% of children attend a facility for early childhood education and care before entering elementary school.^15^ Japanese nursery centers accredited by local governments (those that meet the standards of the Child Welfare Law and related guidelines) provide care for children whose parents are unable to take care of them at home due to work or other reasons. Between 2020 and 2021, the Japanese government declared a state of emergency 4 times. The surveyed municipality closed all nursery centers from mid-April to mid-June 2020 and requested that parents refrain from sending their children to nursery centers. According to our questionnaire for parents, the participating children missed 1.9 days per week of nursery attendance during the emergency periods, while they usually attended 5.1 days per week on average.

**Figure 1.**
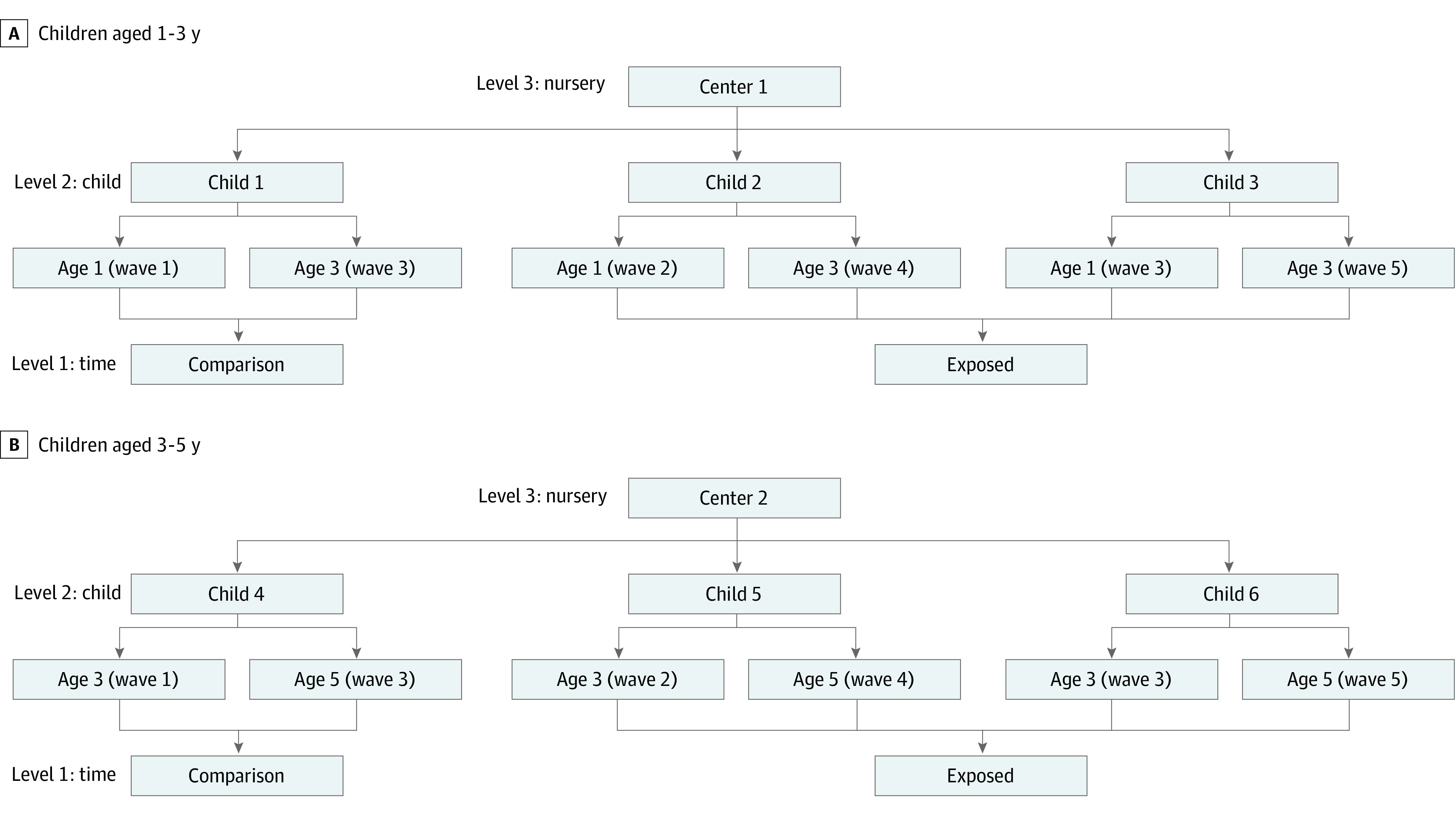
Data Structure Wave 1 (November 2017 to February 2018), wave 2 (October 2018 to January 2019), and wave 3 (October 2019) were conducted before the COVID-19 pandemic, while wave 4 (December 2020 to February 2021) and wave 5 (September 2021 to November 2021) were conducted during the pandemic.

We conducted annual censuses for 5 years (2017-2021) (Figure 1). Waves 1 to 3 were conducted before the COVID-19 outbreak, while waves 4 and 5 were conducted during the pandemic. From the surveys, we constructed 2 sets of panel data comprising children who participated in a baseline survey at age 1 year and were followed up at age 3 years and children who were studied at age 3 years and followed up at age 5 years (henceforth, referred to as the 1-to-3 and 3-to-5 age groups, respectively). Each age group comprised 1 comparison cohort and 2 exposed cohorts; the former was studied in waves 1 and 3 and thus not exposed to the pandemic throughout the study period, and the latter were studied in waves 2 and 4 or waves 3 and 5 and thus exposed to the pandemic at age 3 or 5 years.

Although we invited all accredited nursery centers in the municipality, 5 centers did not participate in wave 1, and 2 centers did not participate in wave 2. Among 1000 children aged 1 year and 922 children aged 3 years, baseline survey data with valid responses were collected for 724 (72.4%) and 578 (62.7%), respectively. In 2 years, we successfully followed up with 447 (61.7%) children aged 3 years and 440 (76.1%) aged 5 years (sample flowcharts shown in eFigures 1 and 2 in Supplement 1). Written informed consent was obtained from parents for participation in each wave. This study was reviewed and approved by the Keio University Shonan Fujisawa Campus Ethics Committee for Experiments and Research (No. 173). The study followed the Strengthening the Reporting of Observational Studies in Epidemiology (STROBE) reporting guideline.^16^

### Outcome

The child’s developmental age was measured using the Kinder Infant Development Scale (KIDS), which was developed using data from 6090 Japanese children.^17^ KIDS was correlated with the Stanford-Binet Intelligence Scales (*r* = 0.86) and the Wechsler Preschool and Primary Scale of Intelligence (*r* = 0.65)^18^ and has been validated in other studies.^19,20^ The parent questionnaires asked whether the child was capable of certain behaviors categorized into 8 domains: physical motor, manipulation, receptive language, expressive language, language concepts, social relationships with children, social relationships with adults, and discipline. The questionnaire comprised 142 items for children younger than 3 years and 133 for children older than 3 years (eMethods in Supplement 1). Among the children, the items indicated high consistency for all ages (Cronbach α, .96-.97 for the overall score) (eTable 1 in Supplement 1). In this study, nursery center teachers answered whether each child was capable of the behaviors; thereafter, the child’s developmental age in months was determined based on the number of items they were capable of achieving. We examined the comprehensive development and the development of the 8 domains.

### Exposures

Our primary exposure was the COVID-19 pandemic. We created a binary variable indicating 1 if an observation was obtained in waves 4 or 5 and 0 otherwise. We confirmed with a municipal official that there were no changes in policies and regulations that would affect child development between waves 3 and 4 except for responses to the pandemic.

Given that the ecologic system theory highlights the importance of the microsystem and mesosystem (ie, environments in family and nursery and their interactions) in early childhood development,^1,2^ we explored the quality of care at nursery centers and parental depression as exposures. The quality of care was assessed using the Infant/Toddler Environment Rating Scale Revised Edition (ITERS-R)^21^ for children aged 1 year and the Early Childhood Environment Rating Scale, Third Edition (ECERS-3)^22^ for children aged 3 years. The scales evaluate both process and structural quality from aspects such as space and furnishings, personal care routines, language and literacy, learning activities, interaction, and program structure. The score is determined based on 39 and 35 items in the ITERS-R and ECERS-3, respectively, ranging between 1 (the worst quality) and 7 (the best quality). In this study, 2 or 3 trained assessors visited each nursery center and scored the process and structural quality at baseline (details are provided elsewhere^23^). We centered the scores on the mean to be used in the analysis. Parental depression was assessed using the 5-item World Health Organization Wellbeing Index^24^ using a parent self-report questionnaire, with scores ranging from 0 to 25. We considered parents with a score less than 13 as possibly exhibiting depression.^25^

### Other Covariates

We fitted the child’s age to a spline function every 4 months. Furthermore, our models included children’s sex and family household income categorized into 3 levels: low (the amount of municipal tax based on household income was <133 000 Japanese yen [JPY]), middle (133 000-301 000 JPY), and high (>301 000 JPY). These covariates were obtained from municipality-provided administrative data. The following variables were collected using a parent self-report questionnaire: child born with a low birth weight (<2500 g), single parent, having a sibling, mother’s working status, father’s working status, and number of days spent at a nursery center per week.

### Statistical Analysis

Linear mixed-effects models were fitted to examine the association between the pandemic and child development (eMethods in Supplement 1). The data had a 3-level hierarchical structure; observations of 2 waves at level 1 were nested within children at level 2 and within nursery centers at level 3. The multilevel model included exposures and covariates in the fixed parts and random intercepts and slopes of the binary variable indicating the pandemic in levels 2 and 3 of the random parts. The random slopes represent additional between-child and between-nursery variations in child development during the pandemic (ie, random parts not explained by included exposures and covariates). We also examined effect modification by different levels in care quality at nursery centers, parental depression, and sex.

We compared the development of children between the exposed and comparison cohorts, but the baseline characteristics differed by cohort. Furthermore, some participants failed to follow up. We calculated the propensity scores of being in the exposed cohort and being followed up and adjusted them as covariates to mitigate potential sample selection bias and attrition bias (eMethods in Supplement 1). Missing values were imputed using a random forests–based algorithm,^26^ assuming that the data were missing at random. Linear mixed-effects models were fitted using the Markov chain Monte Carlo method. Analyses were performed using R, version 4.2.2 statistical software (R Foundation for Statistical Computing); MLwiN, version 3.05 (Centre for Multilevel Modeling, University of Bristol); and Stata, version 18.0 (StataCorp LLC).

## Results

The sample included 447 children in the 1-to-3 age group (201 girls [45.0%] and 246 boys [55.0%]) and 440 children in the 3-to-5 age group (200 girls [45.5%] and 240 boys [54.5%]). The mean (SD) ages at follow-up for the exposed cohorts were 50.0 (3.6) months for the 1-to-3 age group and 73.7 (3.5) months for the 3-to-5 age group. The baseline characteristics differed between the exposed and comparison cohorts (Table). After weighting by the inverse of the propensity score, however, the characteristics of both cohorts were well balanced except for the variables of siblings and father’s working status among children in the 3-to-5 age group (eTables 2 and 3 in Supplement 1). These covariates were also controlled in the regression model. We also adopted propensity scores to address potential attrition bias and confirmed that the characteristics were balanced between those who were successfully followed up and those who were lost to follow-up in both age groups after weighting (eTables 4 and 5 in Supplement 1). Developmental distributions were wider among the 3-to-5 age group and during the pandemic (eFigures 3 and 4 in Supplement 1).

**Table.** Characteristics of Participants

Characteristic	No. (%)							
	1- to 3-y age group (n = 447)				3- to 5-y age group (n = 440)			
	Exposed (n = 323)		Comparison (n = 124)		Exposed (n = 349)		Comparison (n = 91)	
	Baseline (age 1 y)	Follow-up (age 3 y)	Baseline (age 1 y)	Follow-up (age 3 y)	Baseline (age 3 y)	Follow-up (age 5 y)	Baseline (age 3 y)	Follow-up (age 5 y)
Age, mo, mean (SD)	25.2 (3.4)	50.0 (3.6)	28.0 (3.5)	49.0 (3.4)	49.0 (3.3)	73.7 (3.5)	52.3 (3.4)	73.1 (3.4)
Sex								
Female	145 (44.9)	NA	56 (45.2)	NA	158 (45.3)	NA	42 (46.2)	NA
Male	178 (55.1)	NA	68 (54.8)	NA	191 (54.7)	NA	49 (53.8)	NA
Low birth weight	25 (7.7)	NA	14 (11.3)	NA	29 (8.3)	NA	6 (6.6)	NA
Single parent	12 (3.7)	17 (5.3)	9 (7.3)	11 (8.9)	23 (6.6)	21 (6.0)	4 (4.4)	6 (6.6)
Having a sibling	165 (51.1)	224 (69.3)	51 (41.1)	74 (59.7)	229 (65.6)	262 (75.1)	61 (67.0)	66 (72.5)
Working mother	314 (97.2)	308 (95.4)	120 (96.8)	119 (96.0)	335 (96.0)	342 (98.0)	89 (97.8)	89 (97.8)
Working father	310 (96.0)	306 (94.7)	115 (92.7)	112 (90.3)	326 (93.4)	330 (94.6)	87 (95.6)	85 (93.4)
Household income								
Low	66 (20.4)	40 (12.4)	36 (29.0)	21 (16.9)	58 (16.6)	47 (13.5)	18 (19.8)	12 (13.2)
Middle	191 (59.1)	155 (48.0)	70 (56.5)	53 (42.7)	161 (46.1)	144 (41.3)	40 (44.0)	44 (48.4)
High	66 (20.4)	128 (39.6)	18 (14.5)	50 (40.3)	130 (37.2)	158 (45.3)	33 (36.3)	35 (38.5)
Days at nursery, mean (SD)	5.2 (0.4)	5.1 (0.4)	5.1 (0.4)	5.1 (0.3)	5.1 (0.4)	5.1 (0.4)	5.0 (0.3)	5.1 (0.3)
Parental depression	90 (27.9)	78 (24.1)	33 (26.6)	28 (22.6)	86 (24.6)	83 (23.8)	26 (28.6)	27 (29.7)
Quality of care score, mean (SD)^a^	4.4 (0.8)	NA	4.0 (0.6)	NA	3.4 (0.6)	NA	2.9 (0.7)	NA
KIDS, mo, mean (SD)								
Overall	24.1 (4.6)	51.7 (7.9)	24.3 (4.3)	49.4 (7.4)	50.3 (7.1)	66.2 (9.7)	52.1 (7.2)	69.2 (10.4)
Physical motor	24.2 (5.7)	44.6 (7.4)	23.6 (5.4)	41.9 (4.4)	43.2 (5.6)	59.1 (11.7)	45.2 (7.5)	63.1 (12.1)
Manipulation	24.7 (5.5)	55.0 (10.7)	25.2 (5.1)	51.7 (9.7)	53.0 (9.7)	68.2 (10.5)	54.3 (8.7)	70.5 (10.4)
Receptive language	25.7 (7.1)	50.6 (8.3)	26.9 (7.5)	50.1 (9.7)	49.8 (9.0)	68.2 (9.8)	52.2 (9.5)	69.5 (11.3)
Expressive language	24.6 (5.3)	49.5 (8.1)	26.1 (5.4)	48.9 (9.6)	48.5 (8.4)	65.4 (11.3)	49.6 (8.0)	69.4 (12.6)
Language concepts	24.3 (6.9)	54.2 (11.7)	24.7 (6.7)	49.9 (11.2)	52.0 (11.6)	72.6 (11.1)	53.3 (13.0)	72.5 (11.9)
Social relationships with children	22.0 (4.8)	53.3 (8.1)	21.7 (4.2)	49.9 (7.0)	51.7 (7.4)	61.5 (8.4)	54.4 (6.3)	63.0 (8.5)
Social relationships with adults	21.3 (6.0)	53.0 (12.9)	21.0 (5.4)	49.7 (10.8)	51.4 (10.8)	62.9 (15.8)	52.2 (10.6)	68.7 (15.2)
Discipline	22.4 (4.3)	49.8 (10.1)	22.3 (4.2)	49.7 (9.2)	49.4 (8.8)	63.7 (13.7)	50.5 (9.4)	68.5 (12.5)

Abbreviations: KIDS, Kinder Infant Development Scale; NA, not applicable.

^a^ The quality of care at nursery centers was measured using the Infant/Toddler Environment Rating Scale for the 1-to-3 age group and the Early Childhood Environment Rating Scale, Third Edition for the 3-to-5 age group. Imputed data were used. Higher scores indicate better quality.

**Figure 2.**
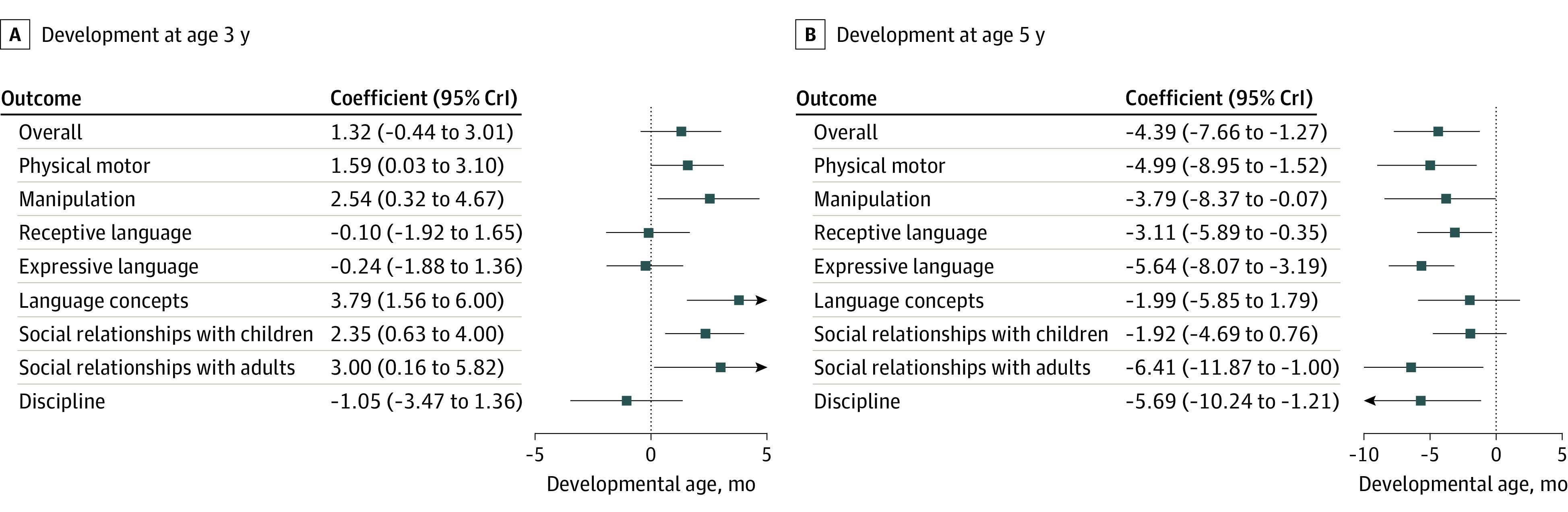
Association Between the Pandemic and Child Development All models were adjusted for the quality of care, parental depression, child’s sex, low birth weight, single parent, sibling, working status of mother and father, household income, number of days in the nursery, child’s age, and a propensity score. CrI indicates credible interval.

In the 1-to-3 age group, the exposed cohort did not show a negative association between the pandemic and overall development (coefficient, 1.32; 95% credible interval, −0.44 to 3.01). Rather, the associations were positive in physical motor, manipulation, language concepts, and social relationships with children and adults (Figure 2; model 2 in eTables 6-14 in Supplement 1). In contrast, in the 3-to-5 age group, the exposed cohorts were 4.39 months behind in overall development at age 5 vs the comparison cohorts (coefficient, −4.39; 95% credible interval, −7.66 to −1.27). Additionally, all of the subdomains indicated negative associations with the pandemic (Figure 2; model 2 in eTables 15-23 in Supplement 1).

**Figure 3.**
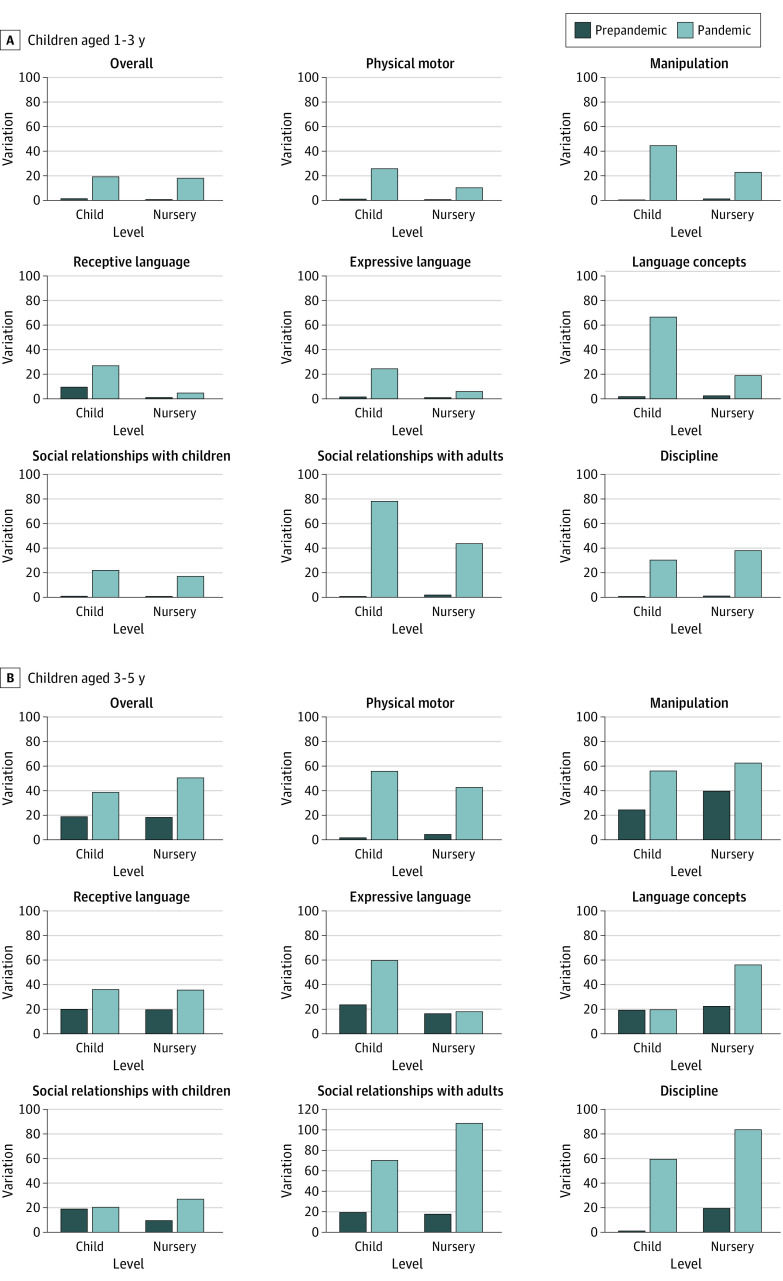
Changes in Between-Child and Between-Nursery Variations in Child Development All models were adjusted for the quality of care, parental depression, child’s sex, low birth weight, single parent, sibling, working status of mother and father, household income, number of days in the nursery, child’s age, and a propensity score.

Figure 3 summarizes the random effects at levels 2 and 3 and shows the changes in between-child and between-nursery variations in child development. During the pandemic, for the 1-to-3 age group, the variations in overall development were approximately 8 and 16 times greater across children and nursery centers, respectively, while variations for the 3-to-5 age group were approximately 2 and 3 times greater across children and nursery centers, respectively. In both age groups, social relationships with adults showed the greatest variation during the pandemic.

**Figure 4.**
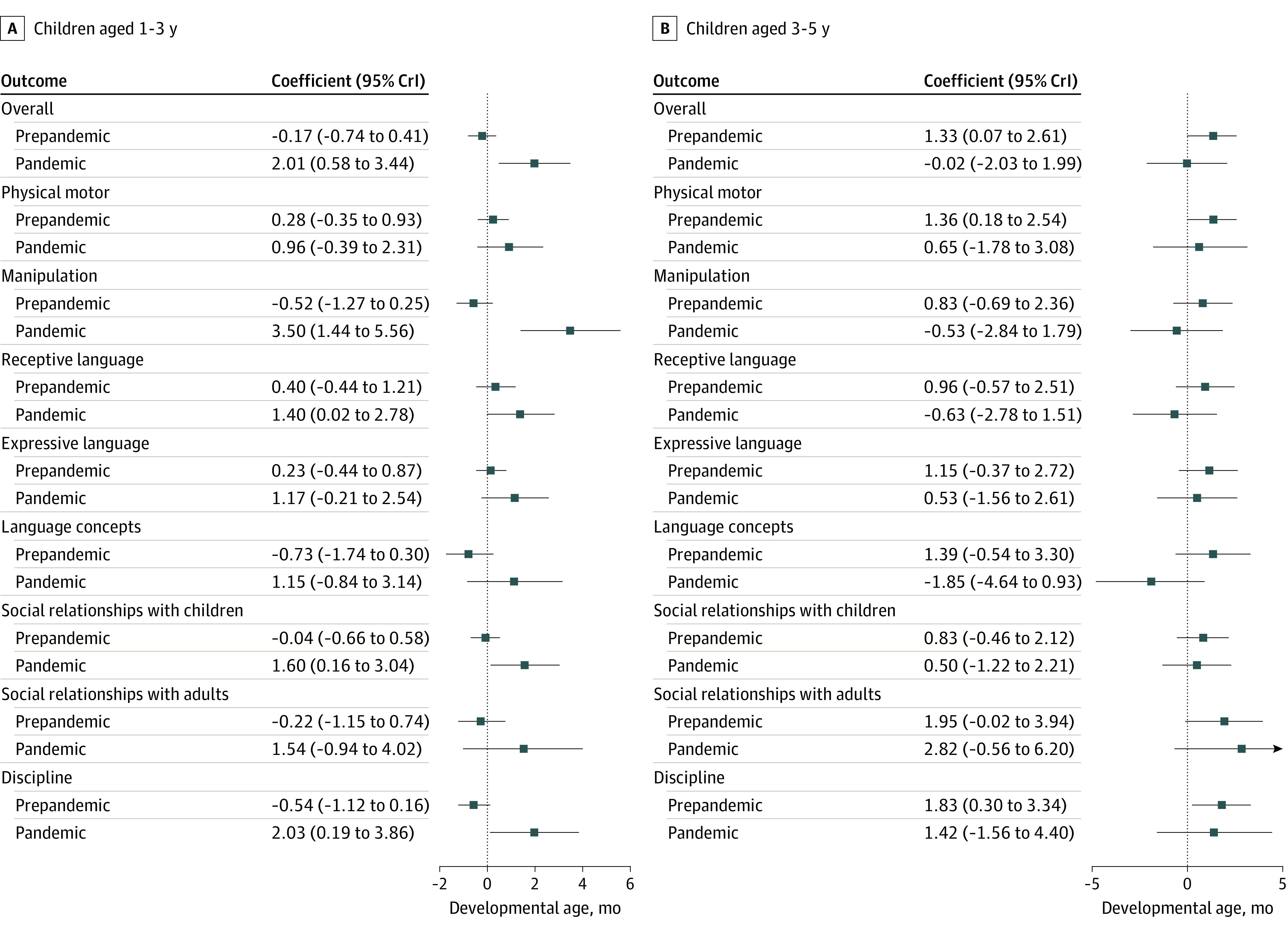
Marginal Association Between the Nursery’s Quality of Care and Child Development All models were adjusted for the quality of care, parental depression, child’s sex, low birth weight, single parent, sibling, working status of mother and father, household income, number of days in the nursery, child’s age, and a propensity score. CrI indicates credible interval.

The nursery center’s quality of care was positively associated with overall development at age 3 years during the pandemic (coefficient, 2.01; 95% credible interval, 0.58-3.44), and all of the subdomains also showed positive associations (Figure 4). In contrast, parental depression appeared to amplify the negative association between the pandemic and overall development at age 5 years during the pandemic (coefficient of interaction, −2.62; 95% credible interval, −4.80 to −0.49; *P* = .009) (model 4 in eTable 15 in Supplement 1; eFigure 5B in Supplement 1), and similar trends were observed in the subdomains (eFigure 5B in Supplement 1).

We found that the associations between the pandemic and child development were almost homogeneous by sex except for manipulation in the 1-to-3 age group, where girls exhibited a more positive association than boys (eFigure 6 in Supplement 1). We also performed several sensitivity analyses and confirmed the robustness of our findings (eMethods, eTable 24, and eFigures 7-11 in Supplement 1).

## Discussion

In this cohort study, we examined the association between the COVID-19 pandemic and early childhood development in a single municipality in Japan. We found that the exposed cohorts were 4.39 months behind in overall development at age 5 years. Such a negative association was not observed in development at age 3 years.

The negative estimate at age 5 years was large. Given that the mean (SD) age at follow-up for the exposed cohorts was 73.7 (3.5) months, the estimate of 4.39 months corresponded to a 6% delay compared with typical development. To compare with another setting, children in primary schools that were highly affected by a major bushfire in Australia indicated approximately 5.5% lower scores in reading and numeracy tests than children in schools that were not affected.^27^ With age, developmental differences across children magnify, and communication with non–family members becomes pivotal in their development.^1,2,28^ Reduced communication with peers and teachers may have particularly affected the cohorts that were exposed to the pandemic at age 5 years.

In contrast, in the 1-to-3 age group, the point estimates associated with the pandemic tended to be positive. Although evidence of an association between day care use and infants’ development is mixed, the association can be negative in specific settings, such as a household with high socioeconomic status. A previous study in a high-income population showed that attendance at day care facilities at ages 0 to 2 years was associated with a reduced intelligence quotient in children aged 8 to 14 years.^29^ Our study was also conducted in a high-income population, with findings consistent with this previous study. Psychological theories emphasize the importance of 1-to-1 interactions between an infant and adults in the child’s development.^30,31,32,33^ The pandemic increased the amount of time parents stayed at home, so increased 1-to-1 interactions within the family may have offset the negative outcomes of the pandemic among the exposed cohorts at age 3 years.

Our study findings also revealed greater variations in child development during the pandemic at both the child and nursery center level. Previous studies suggested that participation in day care is beneficial for the development of children from low-income families.^34,35,36^ Thus, refraining from attending nursery centers may have had a disproportionate association for children from low-income families and may have widened development variations. While the variations widened across almost all developmental domains, social relationships with adults varied the most in both age groups. Social distancing measures have changed the social environment surrounding children, resulting in greater social skill variation. Additionally, nursery centers responded differently to the pandemic by restricting activities and canceling events, which may have generated variations at the nursery level.^13^

Furthermore, our findings show that a higher quality of care provided by a nursery center was positively associated with development during the pandemic, especially at age 3 years. This finding is in line with previous studies that showed that the quality of care at early childcare facilities may be associated with the cognitive and social competencies of children aged up to 54 months.^37,38^ We also found that parental depression amplified the associations between the pandemic and delayed development. Early childcare facilities could mitigate the detrimental association between maternal depression and child developmental problems.^39^ However, during the pandemic, parents had to refrain from sending their children to nursery centers, thus reducing the buffer function of the facility. As a previous study showed that longer exposure to parental mental health problems was associated with greater distress in children,^40^ the association may have been more evident at age 5 years than at age 3 years.

### Limitations

This study had several limitations. First, the response and follow-up rates were moderate (62%-76%). Although we addressed potential attrition bias using propensity scores, we could not obtain information on children whose parents did not respond to the baseline survey. If low-income families were unable to participate in the baseline survey because of pandemic restrictions, our results could be underestimated. Second, we could not infer strict causality from our findings. Although the baseline characteristics were well balanced between the exposed and comparison cohorts based on propensity scores, the estimated association between the pandemic and child development could be confounded by unobserved cohort effects. However, our findings seemed to provide the best knowledge because the pandemic affected the entire population, making it impossible to randomize exposure to the pandemic. Third, the generalizability of our findings is limited. Given the admission criteria for Japanese nursery centers, both parents were working in most participating families. Furthermore, participants were limited to residents of a single municipality in Japan. The average income in the municipality exceeds the national average and thus may not represent children in rural areas. Additionally, the Japanese government took relatively generous preventive measures against COVID-19; these did not entail any legally enforceable stipulations and only requested voluntary nursery center closures. In countries that took more stringent measures, the pandemic might have had a greater association with child development than in Japan. Further studies in other settings are thus required to confirm our findings. Fourth, it should be noted that KIDS is a screening tool, though with good validity and reliability, and we used objective scores measured by nursery center teachers. To accurately assess a child’s development, an individualized examination by a specialist is necessary. Nevertheless, KIDS provides an informative overview of child development, including typical development. Fifth, the results may be prone to multiple testing problems given that we examined outcomes of 8 developmental domains. Readers should look at overall patterns of association rather than the statistical significance of individual associations.

## Conclusions

The findings of this cohort study show an association between exposure to the pandemic and delayed development at age 5 years. Variations in development widened during the pandemic regardless of age. Particularly, social relationships with adults appeared to drive the increased variability. Additionally, the quality of care at nursery centers was positively associated with development at age 3 years during the pandemic, while parental depression appeared to amplify the association between the pandemic and delayed development at age 5 years. It is important to identify children who have been detrimentally affected by the pandemic and provide them with support for learning, socialization, physical and mental health, and family support.

## Notes

Supplement 1. **eFigure 1.** Sample Flowchart for 1 to 3 Years Old **eFigure 2.** Sample Flowchart for 3 to 5 Years Old
eMethods
**eTable 1.** Cronbach’s Alpha for the Kinder Infant Development Scale **eTable 2.** Balance Check Between Exposed and Comparison Cohorts for 1 to 3 Years Old **eTable 3.** Balance Check Between Exposed and Comparison Cohorts for 3 to 5 Years Old **eTable 4.** Balance Check Between Those Who Were Followed Up and Lost to Follow-up for 1 to 3 Years Old **eTable 5.** Balance Check Between Those Who Were Followed Up and Lost to Follow-up for 3 to 5 Years Old **eFigure 3.** Changes in the Outcome Scores and Distributions for 1 to 3 Years Old **eFigure 4.** Changes in the Outcome Scores and distributions for 3 to 5 Years Old **eTable 6.** Association Between the Pandemic and Overall Development for 1 to 3 Years Old **eTable 7.** Association Between the Pandemic and Physical Motor for 1 to 3 Years Old **eTable 8.** Association Between the Pandemic and Manipulation for 1 to 3 Years Old **eTable 9.** Association Between the Pandemic and Receptive Language for 1 to 3 Years Old **eTable 10.** Association Between the Pandemic and Expressive Language for 1 to 3 Years Old **eTable 11.** Association Between the Pandemic and Language Concepts for 1 to 3 Years Old **eTable 12.** Association Between the Pandemic and Social Relationships With Children for 1 to 3 Years Old **eTable 13.** Association Between the Pandemic and Social Relationships With Adults for 1 to 3 Years Old **eTable 14.** Association Between the Pandemic and Discipline for 1 to 3 Years Old **eTable 15.** Association Between the Pandemic and Overall Development for 3 to 5 Years Old **eTable 16.** Association Between the Pandemic and Physical Motor for 3 to 5 Years Old **eTable 17.** Association Between the Pandemic and Manipulation for 3 to 5 Years Old **eTable 18.** Association Between the Pandemic and Receptive Language for 3 to 5 Years Old **eTable 19.** Association Between the Pandemic and Expressive Language for 3 to 5 Years Old **eTable 20.** Association Between the Pandemic and Language Concepts for 3 to 5 Years Old **eTable 21.** Association Between the Pandemic and Social Relationships With Children for 3 to 5 Years Old **eTable 22.** Association Between the Pandemic and Social Relationships With Adults for 3 to 5 Years Old **eTable 23.** Association Between the Pandemic and Discipline for 3 to 5 Years Old **eFigure 5.** Marginal Association Between Parental Depression and Child Development **eFigure 6.** Marginal Association Between Sex and Child Development **eFigure 7.** Association Between the Pandemic and Child Development Without Adjustment of Propensity Scores **eFigure 8.** Association between the Pandemic and Child Development Nested Within the Nursery at Follow-up **eTable 24.** Number of Missing Values **eFigure 9.** Complete Case Analysis for the Association Between the Pandemic and Child Development **eFigure 10.** Subgroup Analysis by Follow-up Timing for the Association Between the Pandemic and Child Development **eFigure 11.** Association Between the Pandemic and Child Development Using a Saturated Function for Age

Supplement 2.
Data Sharing Statement
