# Supplementary material for: Association Between the COVID-19 Pandemic and Early Childhood Development
Source: JAMA Pediatr. 2023 Jul 10;177(9):930–8. doi: 10.1001/jamapediatrics.2023.2096 (PMC10334298; doi:10.1001/jamapediatrics.2023.2096)
Supplement: Supplement 1. — eFigure 1. Sample Flowchart for 1 to 3 Years Old eFigure 2. Sample Flowchart for 3 to 5 Years Old eMethods eTable 1. Cronbach’s Alpha for the Kinder Infant Development Scale eTable 2. Balance Check Between Exposed and Comparison Cohorts for 1 to 3 Years Old eTable 3. Balance Check Between Exposed and Comparison Cohorts for 3 to 5 Years Old eTable 4. Balance Check Between Those Who Were Followed Up and Lost to Follow-up for 1 to 3 Years Old eTable 5. Balance Check Between Those Who Were Followed Up and Lost to Follow-up for 3 to 5 Years Old eFigure 3. Changes in the Outcome Scores and Distributions for 1 to 3 Years Old eFigure 4. Changes in the Outcome Scores and distributions for 3 to 5 Years Old eTable 6. Association Between the Pandemic and Overall Development for 1 to 3 Years Old eTable 7. Association Between the Pandemic and Physical Motor for 1 to 3 Years Old eTable 8. Association Between the Pandemic and Manipulation for 1 to 3 Years Old eTable 9. Association Between the Pandemic and Receptive Language for 1 to 3 Years Old eTable 10. Association Between the Pandemic and Expressive Language for 1 to 3 Years Old eTable 11. Association Between the Pandemic and Language Concepts for 1 to 3 Years Old eTable 12. Association Between the Pandemic and Social Relationships With Children for 1 to 3 Years Old eTable 13. Association Between the Pandemic and Social Relationships With Adults for 1 to 3 Years Old eTable 14. Association Between the Pandemic and Discipline for 1 to 3 Years Old eTable 15. Association Between the Pandemic and Overall Development for 3 to 5 Years Old eTable 16. Association Between the Pandemic and Physical Motor for 3 to 5 Years Old eTable 17. Association Between the Pandemic and Manipulation for 3 to 5 Years Old eTable 18. Association Between the Pandemic and Receptive Language for 3 to 5 Years Old eTable 19. Association Between the Pandemic and Expressive Language for 3 to 5 Years Old eTable 20. Association Between the Pandemic and Language Conc [file jamapediatr-e232096-s001.pdf]

Supplemental Online Content

Sato K, Fukai T, Fujisawa KK, Nakamuro M. Association between the COVID-19 pandemic and early childhood development. *JAMA Pediatr*. Published online July 10, 2023. doi:10.1001/jamapediatrics.2023.2096

eFigure 1. Sample Flowchart for 1 to 3 Years Old

eFigure 2. Sample Flowchart for 3 to 5 Years Old

eMethods

eTable 1. Cronbach's Alpha for the Kinder Infant Development Scale

eTable 2. Balance Check Between Exposed and Comparison Cohorts for 1 to 3 Years Old

eTable 3. Balance Check Between Exposed and Comparison Cohorts for 3 to 5 Years Old

eTable 4. Balance Check Between Those Who Were Followed Up and Lost to Follow-up for 1 to 3 Years Old

eTable 5. Balance Check Between Those Who Were Followed Up and Lost to Follow-up for 3 to 5 Years Old

eFigure 3. Changes in the Outcome Scores and Distributions for 1 to 3 Years Old

eFigure 4. Changes in the Outcome Scores and distributions for 3 to 5 Years Old

eTable 6. Association Between the Pandemic and Overall Development for 1 to 3 Years Old

eTable 7. Association Between the Pandemic and Physical Motor for 1 to 3 Years Old

eTable 8. Association Between the Pandemic and Manipulation for 1 to 3 Years Old

eTable 9. Association Between the Pandemic and Receptive Language for 1 to 3 Years Old

eTable 10. Association Between the Pandemic and Expressive Language for 1 to 3 Years Old

eTable 11. Association Between the Pandemic and Language Concepts for 1 to 3 Years Old

eTable 12. Association Between the Pandemic and Social Relationships With Children for 1 to 3 Years Old

eTable 13. Association Between the Pandemic and Social Relationships With Adults for 1 to 3 Years Old

eTable 14. Association Between the Pandemic and Discipline for 1 to 3 Years Old

© 2023 Sato K et al. *JAMA Pediatrics.*

eTable 15. Association Between the Pandemic and Overall Development for 3 to 5 Years Old

eTable 16. Association Between the Pandemic and Physical Motor for 3 to 5 Years Old

eTable 17. Association Between the Pandemic and Manipulation for 3 to 5 Years Old

eTable 18. Association Between the Pandemic and Receptive Language for 3 to 5 Years Old

eTable 19. Association Between the Pandemic and Expressive Language for 3 to 5 Years Old

eTable 20. Association Between the Pandemic and Language Concepts for 3 to 5 Years Old

eTable 21. Association Between the Pandemic and Social Relationships With Children for 3 to 5 Years Old

eTable 22. Association Between the Pandemic and Social Relationships With Adults for 3 to 5 Years Old

eTable 23. Association Between the Pandemic and Discipline for 3 to 5 Years Old

eFigure 5. Marginal Association Between Parental Depression and Child Development

eFigure 6. Marginal Association Between Sex and Child Development

eFigure 7. Association Between the Pandemic and Child Development Without Adjustment of Propensity Scores

eFigure 8. Association between the Pandemic and Child Development Nested Within the Nursery at Follow-up

eTable 24. Number of Missing Values

eFigure 9. Complete Case Analysis for the Association Between the Pandemic and Child Development

eFigure 10. Subgroup Analysis by Follow-up Timing for the Association Between the Pandemic and Child Development

eFigure 11. Association Between the Pandemic and Child Development Using a Saturated Function for Age

This supplemental material has been provided by the authors to give readers additional information about their work.

© 2023 Sato K et al. *JAMA Pediatrics.*

eFigure 1. Sample Flowchart for 1 to 3 Years Old

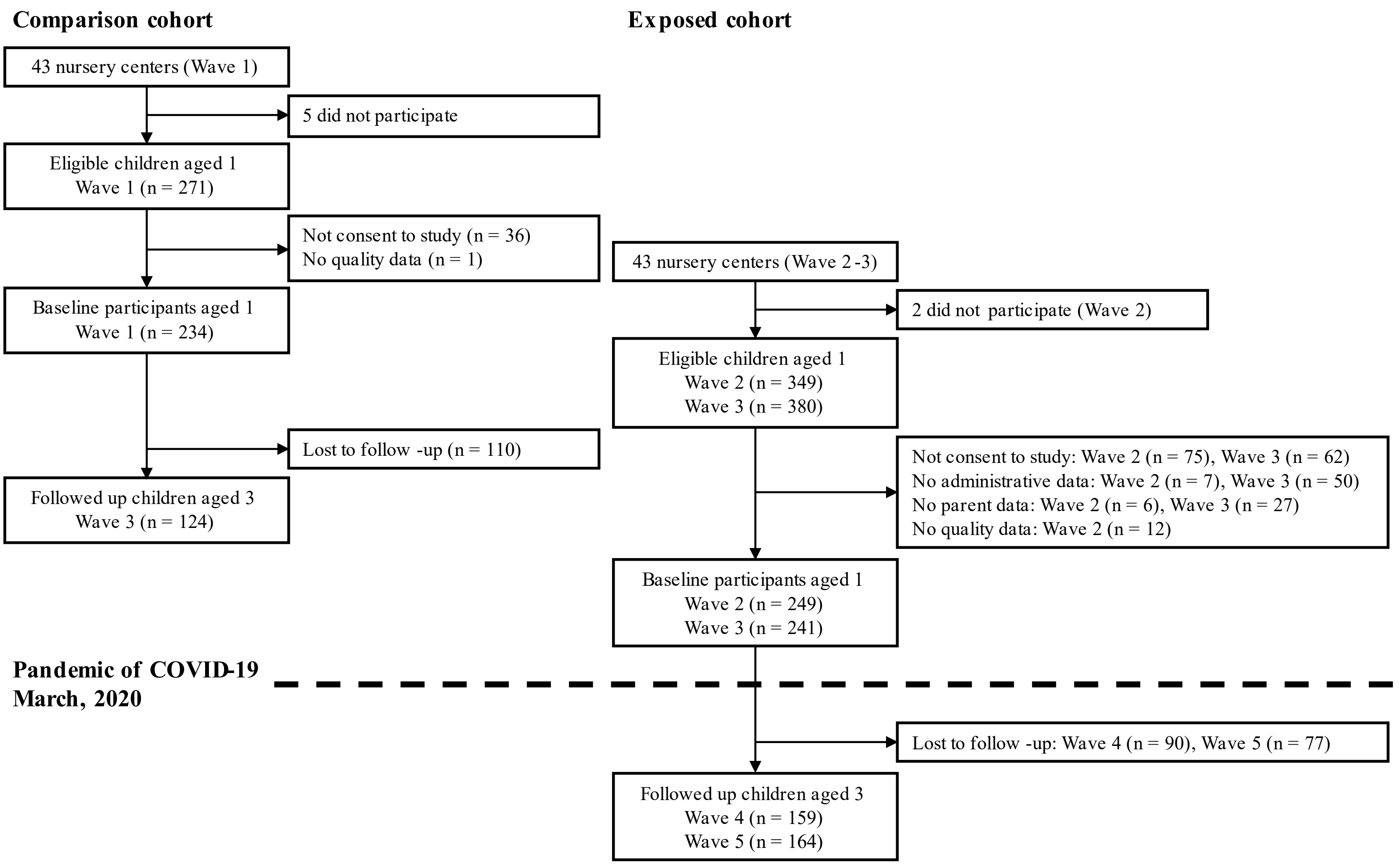

© 2023 Sato K et al. *JAMA Pediatrics.*

eFigure 2. Sample Flowchart for 3 to 5 Years Old

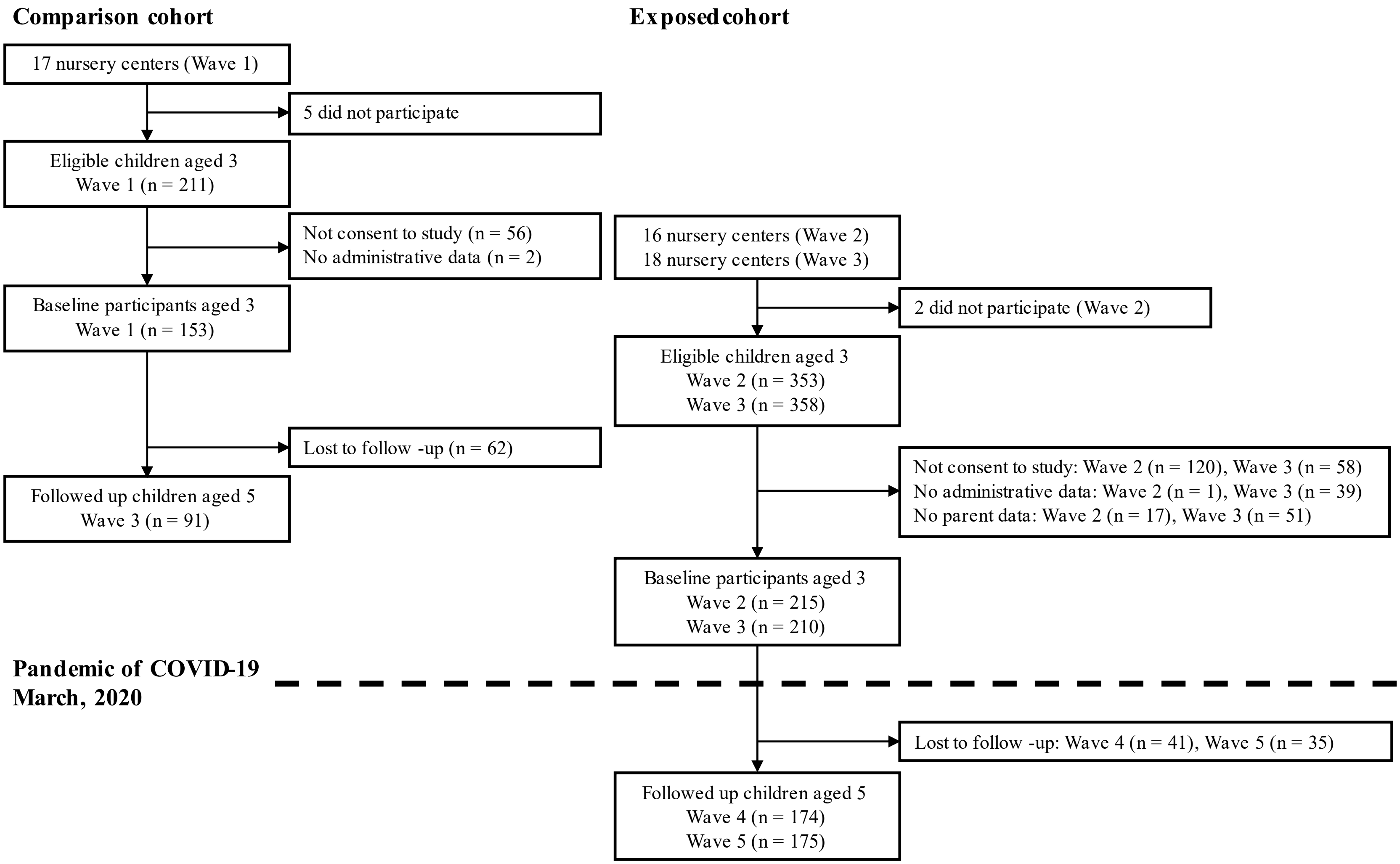

© 2023 Sato K et al. *JAMA Pediatrics.*

eMethods

1. Items in the Kinder Infant Development Scale (KIDS)

Instructions: Please answer the questions as to what a child can or cannot do.
The child …

1) Physical motor

1. Can roll over on the floor.
2. Can slide down a slide on his/her back.
3. Can catch a rolling ball.
4. Can hop on one foot.
5. Can run about 20m smoothly at full speed.
6. Can stand on one leg for about 5 seconds (may move).
7. Can climb to the top of a jungle gym in a park.
8. Can walk on tiptoe.
9. Can stand on a swing.
10. Can skip.
11. Can play relay games by themselves.
12. Can dribble a ball about three times.
13. Can play chase on a jungle gym without touching the ground.
14. Can pedal a swing while standing up.
15. Can jump rope by him/herself.
16. Can ride a bicycle without training wheels.

2) Manipulation

1. Applies glue and stick paper by him/herself.
2. Uses scissors to cut paper into straight lines.
3. Draws a cross shape by imitating.
4. Cut simple shapes with scissors.
5. Draws people, etc.
6. Draws pictures with crayons using different colors.
7. Makes a tunnel through a pile of sand in the sandbox.
8. Draws a car, a flower, or whatever he/she thinks of (it should look like that).
9. Makes a 20-piece jigsaw puzzle.
10. Draws a picture using crayons and paints.
11. Draws a picture of what he/she experienced (it should look like that).
12. Can operate a cassette tape deck.
13. Draws a diamond shape.
14. Draws what he/she hears (it should look like that).
15. Gets interested in maps and globes.
16. Can make origami cranes.

3) Receptive language

1. Knows the names of friends.
2. Knows all the uses of the following items (comb, hat, mirror, cup, and pencil).
3. Memorizes a sentence from a fairy tale or other story heard many times.
4. Can correctly answer the question "What do you do when you are hungry?"
5. Can count to 10.
6. Knows what both eyes and ears do.
7. Can sing at least 10 songs.
8. Knows how many fingers there are.
9. Can read his/her name written in Hiragana.
10. Asks for help when there is a character that he/she does not understand.
11. Can play riddles.
12. Can add up to five or less (1+1, 1+2, etc.).
13. Can tell his/her birthday (date of birth).

© 2023 Sato K et al. *JAMA Pediatrics.*

14. Can read all 46 Hiragana characters.
15. Knows what day of the month it is.
16. Can tell the time on a clock (excluding digital displays).

4) Expressive language
1. Can converse with other children of the same age.
2. Talks to parents about what he/she sees and hears.
3. Talks a lot while playing.
4. Talks happily alone while looking at pictures.
5. Talks with other children of the same age.
6. Can say both parents' names.
7. Pronounces “kya,” “kyu,” and “kyo” clearly.
8. Does not use infantile language.
9. Can talk about yesterday's events.
10. Can correct his/her pronunciation when it is incorrect.
11. Can play “Shiritori (Japanese word chain game).”
12. Can say all the days of the week.
13. Can say three numbers backwards (295, 816, etc.).
14. Reads picture book sentences clearly and aloud.
15. Can explain the way to a place such as a park correctly.
16. Can say tongue twisters.

5) Language concepts
1. Understands “dirty/clean.”
2. Understands “good/bad.”
3. Understands “hard/soft.”
4. Understands “promise.”
5. Understands “strong/weak.”
6. Understands “win/lose.”
7. Understands “thick/thin.”
8. Understands “regret.”
9. Understands “thick/thin.”
10. Understands “right/left.”
11. Understands “kindness.”
12. Understands “success”.
13. Understands “futility.”
14. Understands “courage.”
15. Understands “adventure.”
16. Understands “life.”

6) Social relationships with children
1. Plays a role while playing house.
2. Plays the main character on TV.
3. Waits for his/her turn on swings, etc.
4. Makes a pile in the sandbox with two or more children.
5. Plays hide-and-seek to avoid detection.
6. Plays pretend games with the group as one.
7. Compromises to play within the group.
8. Invites friends to the house.
9. Takes care of the little ones.
10. Understands the rules of tag.
11. Talks secretly with two or three people.
12. Understands which wins in a competition between two teams.
13. Decides an order in a game of rock-paper-scissors.
14. Warns a child who does something prohibited.
15. Exchanges toys such as stickers and dolls with friends.
16. Plays organized games such as baseball.

© 2023 Sato K et al. *JAMA Pediatrics.*

7) Social relationships with adults
1. Asks parents to make crafts that he/she cannot do.
2. Asks for permission to do something.
3. Wants to show what he/she has made.
4. When praised, seeks more praise.
5. Follows instructions when asked to lend other children a toy.
6. Follows instructions of teachers at kindergarten and nursery school.
7. Stops misbehaving as soon as a stranger warns in a park or other place.
8. Watches for traffic lights and crosses a street correctly.
9. Tells parents where he/she is going to play.
10. Says “How much is this?” and asks the price.
11. Can receive change when shopping.
12. If scolded for playing a prank, will not do it again.
13. Bursts into tears when hears a sad story.
14. Can greet people whom he/she meets for the first time.
15. When lost the way, asks for directions.
16. Can buy drinks from a vending machine by him/herself.

8) Discipline
1. Takes off pants and pees by him/herself.
2. Takes off pants and skirt by him/herself.
3. Can rinse mouth out.
4. Does not cry when washing head (may use a shampoo hat).
5. Can use chopsticks.
6. Does not wet his/her pants while playing enthusiastically.
7. Can put on pajamas by him/herself.
8. Can wash him/herself easily.
9. Can put on a jumper or other jackets on his/her own.
10. Goes to the bathroom by himself/herself and rarely needs parental help.
11. Can wait until his/her parents’ permission to eat.
12. Washes his/her face by him/herself.
13. Can fold his/her clothes after taking them off.
14. Can wipe his/her own bottom after urinating by him/herself.
15. Can brush his/her teeth by him/herself.
16. Wipes himself/herself after taking a bath.
17. Takes dishes to the washing place and helps clean up after meals.
18. Goes to the bathroom on his/her own before going to bed.
19. Can wash his/her head by him/herself (may use a shampoo hat).
20. Can wait patiently for 15 minutes when told to wait here.
21. Goes to bed at a fixed time every day.

Note: These items are included in the questionnaire for children over 3 years old. We translated Appendix in Miyake et al. (1990).
Reference: Miyake K, Ohmura M, Takashima M, Yamanouchi S, Hashimoto T, Kobayashi K. A New Test Developmental Screening Scale Kinder Infant Development Scale. Hum Dev Res. 1990;6:147-163.

© 2023 Sato K et al. *JAMA Pediatrics.*

2. Linear mixed-effects model
The association between the pandemic and child development was estimated by fitting the following linear mixed-effects model:

$$y_{tij} = \boldsymbol{\beta_0} + \beta_1 COVID19_{tij} + \boldsymbol{age}'\boldsymbol{\beta_2} + \boldsymbol{agecat}'_{c_k}\boldsymbol{\beta_3} + (\boldsymbol{age} - \boldsymbol{c_k}) * \boldsymbol{agecat}'_{c_k}\boldsymbol{\beta_4} + \boldsymbol{W}'\boldsymbol{\beta_5} + e_{0tij} + u_{0ij} + COVID19_{tij} * u_{1ij} + v_{0j} + COVID19_{tij} * v_{1j}$$

where y_{tij} is the score of the Kinder Infant Development Scale (KIDS) for time *t* of child *i* in nursery *j*. $COVID19_{tij}$ denotes whether the observation was collected during the pandemic or not. We fitted child's age to a spline function every four months. $\boldsymbol{age}$ is a vector of child's age. $\boldsymbol{agecat_{c_k}}$ denotes a matrix of binary variables indicating whether the child's age falls into an age category ranging between c_k and $c_k + 3$ months, where c_k is age at the *k*th spline. For the age category of c_k, $\boldsymbol{\beta_0} + \boldsymbol{\beta_3}$ represents an intercept, and $\boldsymbol{\beta_2} + \boldsymbol{\beta_4}$ represents a slope. $\boldsymbol{W}$ is a matrix of other exposures and covariates.

For random parts of the model, e_{0tij} is an within-child random intercept; u_{0ij} is a between-child random intercept; u_{1ij} is a between-child random slope of the pandemic; v_{0j} is a between-nursery random intercept; and v_{1j} is a between-nursery random slope of the pandemic. Given that σ^2_{u0} and σ^2_{u1} denote the variance of u_{0ij} and u_{1ij}, σ^2_{u0} and $\sigma^2_{u0} + \sigma^2_{u1}$ represent a between-child variation in the KIDS score during the pre-pandemic and pandemic periods, respectively. Likewise, given that σ^2_{v0} and σ^2_{v1} denote the variance of v_{0j} and v_{1j}, σ^2_{v0} and $\sigma^2_{v0} + \sigma^2_{v1}$ represent a between-nursery variation during the pre-pandemic and pandemic periods, respectively.

In Model 1, we only adjusted for child's age. In Model 2 (the main model), we additionally adjusted for the quality of care at nursery centers, parental depression, child's sex, low birth weight, single parent, sibling, working status of mother and father, household income, and the number of Days in nursery. Model 3 was adjusted for covariates in Model 2 plus an interaction term between the pandemic and the quality of care. Model 4 was adjusted for covariates in Model 2 plus an interaction term between the pandemic and parental depression. Model 5 was adjusted for covariates in Model 2 plus an interaction term between the pandemic and sex.

3. Propensity score
To mitigate the potential sample selection bias and attrition bias, we adjusted for the following propensity scores in the regression models:

$$PS = \Pr(T = 1|L) * \Pr(A = 1|L, T)$$

where $T = 1$ if the child was in the exposed cohort and $T = 0$ if in the comparison cohort; $A = 1$ if the child was successfully followed up and $A = 0$ if lost to follow-up; L is observed characteristics at the baseline. Missing baseline characteristics were imputed using a random-forests-based algorithm.

The propensity scores were predicted using logistic regression adjusting for the baseline Kinder Infant Development Scale (KIDS) score, child's sex, low birth weight, single parent, sibling, working status of mother and father, household income, the number of days in nursery, parental depression, and child's age and its logarithm. When we estimated $\Pr(T = 1|L)$ for the 1–3 years-old group, an interaction term between parental depression and child's age was added to balance between the treatment and control groups. When we estimated $\Pr(A = 1|L, T)$ for both age groups, we additionally adjusted for whether the participant was in the exposed or comparison cohort.

In general, a standardized difference of less than 0.1 indicates a balance between the two groups. After weighting by the inverse probability of being in the exposed cohort, the characteristics of the exposed and comparison cohorts were well balanced, except for the variables of sibling and father's working status among the 3–5 years-old group (Supplementary Table 1 and 2). The exposed cohort was more likely to have a sibling and less likely to have their fathers working even after weighting. These variables were controlled in the regression models as well as other covariates. After weighting by the inverse probability of being followed up, the characteristics balanced between those who were successfully followed up and those who were lost to follow in both age groups (Supplementary Table 3 and 4).
4. Sensitivity analyses

© 2023 Sato K et al. *JAMA Pediatrics.*

First, the estimates without adjustment of propensity scores indicated similar results to the main results (eFigure 7). Second, if children changed nursery centers at follow-up, they were nested within the baseline centers because the quality of care was measured at baseline. We confirmed that results did not change even if they were nested within the centers at follow-up (eFigure 8). Third, we performed a complete-case analysis (using only observations without missing values), assuming they were missing completely at random. The number of missing values for each variable is shown in eTable 24. The complete-case analysis showed similar results to the main results (eFigure 9). Fourth, in the main results, we combined the cohort that was followed up in 2020 and the one that was followed up in 2021 into the exposed group. The two cohorts differed in the duration of exposure to the pandemic, and its impact could be inconsistent. Thus, we divided the exposed group into two according to the timing of follow-up and separately compared with the comparison group. Regardless of follow-up timings, the associations showed similar patterns in both subgroups, and their credible intervals were overlapped (eFigure 10). Finally, we also tested a saturated function for age (i.e., included dummy variables for each month of age), which allowed a non-linear relationship between age and development, and found that the results were robust (eFigure 11).

eTable 1. Cronbach's Alpha for the Kinder Infant Development Scale

	Age 1	Age 3	Age 5
Overall	0.97	0.96	0.97
Physical Motor	0.85	0.78	0.86
Manipulation	0.87	0.84	0.84
Receptive Lang.	0.84	0.79	0.84
Expressive Lang.	0.89	0.78	0.82
Lang. Concepts	0.88	0.85	0.87
SRs with Children	0.73	0.87	0.89
SRs with Adults	0.79	0.76	0.85
Discipline	0.76	0.84	0.87

Lang. = Language, SR = social relationship.

© 2023 Sato K et al. *JAMA Pediatrics.*

eTable 2. Balance Check Between Exposed and Comparison Cohorts for 1 to 3 Years Old

	Before weighting			After weighting		
	Exposed (mean)	Comparison (mean)	Standardized difference	Exposed (mean)	Comparison (mean)	Standardized difference
KIDS	24.12	24.35	-0.052	24.08	24.24	-0.036
Girl	0.44	0.45	-0.008	0.45	0.47	-0.047
Low birth weight	0.08	0.11	-0.114	0.09	0.10	-0.055
Single parent	0.04	0.07	-0.158	0.04	0.04	0.003
Sibling	0.51	0.42	0.196	0.49	0.50	-0.035
Working mother	0.97	0.97	0.028	0.97	0.97	-0.003
Working father	0.96	0.93	0.143	0.96	0.96	-0.010
Low income	0.21	0.29	-0.192	0.23	0.27	-0.084
Middle income	0.60	0.56	0.071	0.59	0.60	-0.017
Days in nursery	5.16	5.11	0.128	5.15	5.17	-0.041
Depression	0.28	0.27	0.020	0.27	0.23	0.089
Age	25.24	28.05	-0.815	25.91	26.23	-0.091

KIDS = the Kinder Infant Development Scale. In general, a standardized difference of less than 0.1 indicates a balance between the two groups.

eTable 3. Balance Check Between Exposed and Comparison Cohorts for 3 to 5 Years Old

	Before weighting			After weighting		
	Exposed (mean)	Comparison (mean)	Standardized difference	Exposed (mean)	Comparison (mean)	Standardized difference
KIDS	50.35	52.08	-0.242	50.63	51.06	-0.061
Girl	0.45	0.46	-0.018	0.46	0.46	-0.011
Low birth weight	0.08	0.07	0.065	0.08	0.08	0.001
Single parent	0.07	0.04	0.096	0.06	0.03	0.127
Sibling	0.66	0.67	-0.03	0.66	0.64	0.035
Working mother	0.96	0.98	-0.104	0.96	0.96	0.024
Working father	0.93	0.96	-0.096	0.94	0.97	-0.127
Low income	0.17	0.2	-0.082	0.17	0.15	0.036
Middle income	0.46	0.44	0.044	0.46	0.49	-0.052
Days in nursery	5.11	5.04	0.207	5.1	5.09	0.041
Depression	0.25	0.29	-0.089	0.25	0.24	0.027
Age	49.04	52.32	-0.982	49.54	49.87	-0.099

KIDS = the Kinder Infant Development Scale. In general, a standardized difference of less than 0.1 indicates a balance between the two groups.

© 2023 Sato K et al. *JAMA Pediatrics.*

eTable 4. Balance Check Between Those Who Were Followed Up and Lost to Follow-up for 1 to 3 Years Old

	Before weighting			After weighting		
	Followed up (mean)	Lost to follow (mean)	Standardized difference	Followed up (mean)	Lost to follow (mean)	Standardized difference
KIDS	24.18	24.86	-0.150	24.46	24.49	-0.007
Girl	0.45	0.49	-0.085	0.46	0.46	-0.002
Low birth weight	0.09	0.06	0.089	0.08	0.08	0.000
Single parent	0.05	0.04	0.034	0.04	0.04	0.001
Sibling	0.49	0.44	0.098	0.46	0.46	0.013
Working mother	0.97	0.96	0.04	0.97	0.97	-0.001
Working father	0.95	0.96	-0.027	0.95	0.95	-0.002
Low income	0.23	0.34	-0.253	0.27	0.27	-0.003
Middle income	0.59	0.53	0.104	0.57	0.57	-0.006
Days at nursery	5.15	5.12	0.069	5.14	5.14	0.005
Depression	0.28	0.26	0.042	0.27	0.27	-0.002
Age	26.01	26.31	-0.083	26.14	26.16	-0.007
Exposed cohort	0.73	0.61	0.251	0.68	0.68	0.003

KIDS = the Kinder Infant Development Scale. In general, a standardized difference of less than 0.1 indicates a balance between the two groups.

eTable 5. Balance Check Between Those Who Were Followed Up and Lost to Follow-up for 3 to 5 Years Old

	Before weighting			After weighting		
	Followed up (mean)	Lost to follow (mean)	Standardized difference	Followed up (mean)	Lost to follow (mean)	Standardized difference
KIDS	50.7	51.22	-0.070	50.83	50.92	-0.012
Girl	0.45	0.43	0.040	0.45	0.47	-0.042
Low birth weight	0.08	0.12	-0.145	0.09	0.09	0.009
Single parent	0.06	0.08	-0.072	0.07	0.07	-0.011
Sibling	0.66	0.61	0.105	0.65	0.65	0.004
Working mother	0.96	0.91	0.235	0.95	0.95	0.003
Working father	0.94	0.92	0.072	0.93	0.93	0.009
Low income	0.17	0.25	-0.198	0.19	0.2	-0.014
Middle income	0.46	0.47	-0.028	0.46	0.47	-0.014
Days at nursery	5.1	5.09	0.022	5.09	5.09	0.013
Depression	0.25	0.31	-0.127	0.27	0.28	-0.028
Age	49.72	50.51	-0.225	49.91	49.95	-0.012
Exposed cohort	0.79	0.55	0.533	0.74	0.74	-0.001

KIDS = the Kinder Infant Development Scale. In general, a standardized difference of less than 0.1 indicates a balance between the two groups.

© 2023 Sato K et al. *JAMA Pediatrics.*

eFigure 3. Changes in the Outcome Scores and Distributions for 1 to 3 Years Old

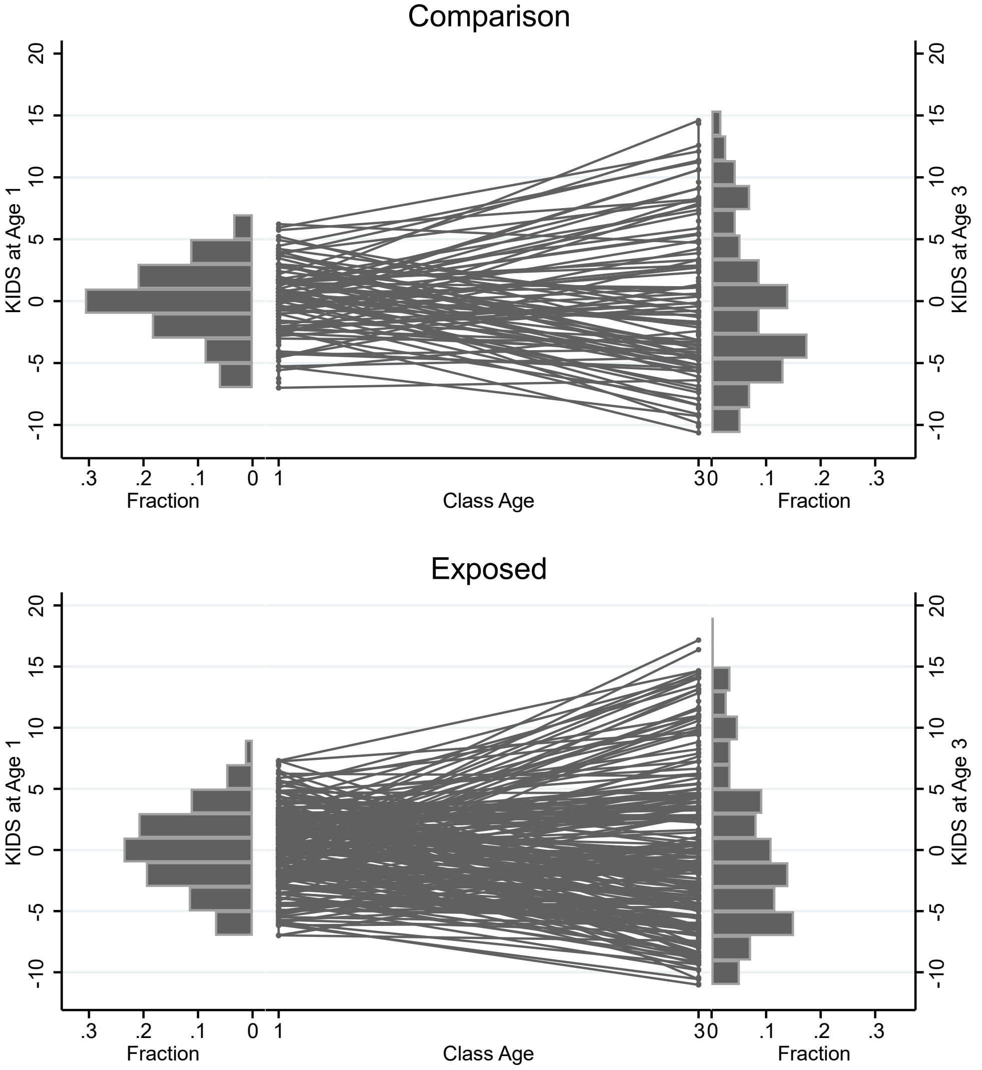

KIDS = the Kinder Infant Development Scale. The KIDS scores were adjusted for age in months, and the score is 0 for typical development.

© 2023 Sato K et al. *JAMA Pediatrics.*

eFigure 4. Changes in the Outcome Scores and distributions for 3 to 5 Years Old

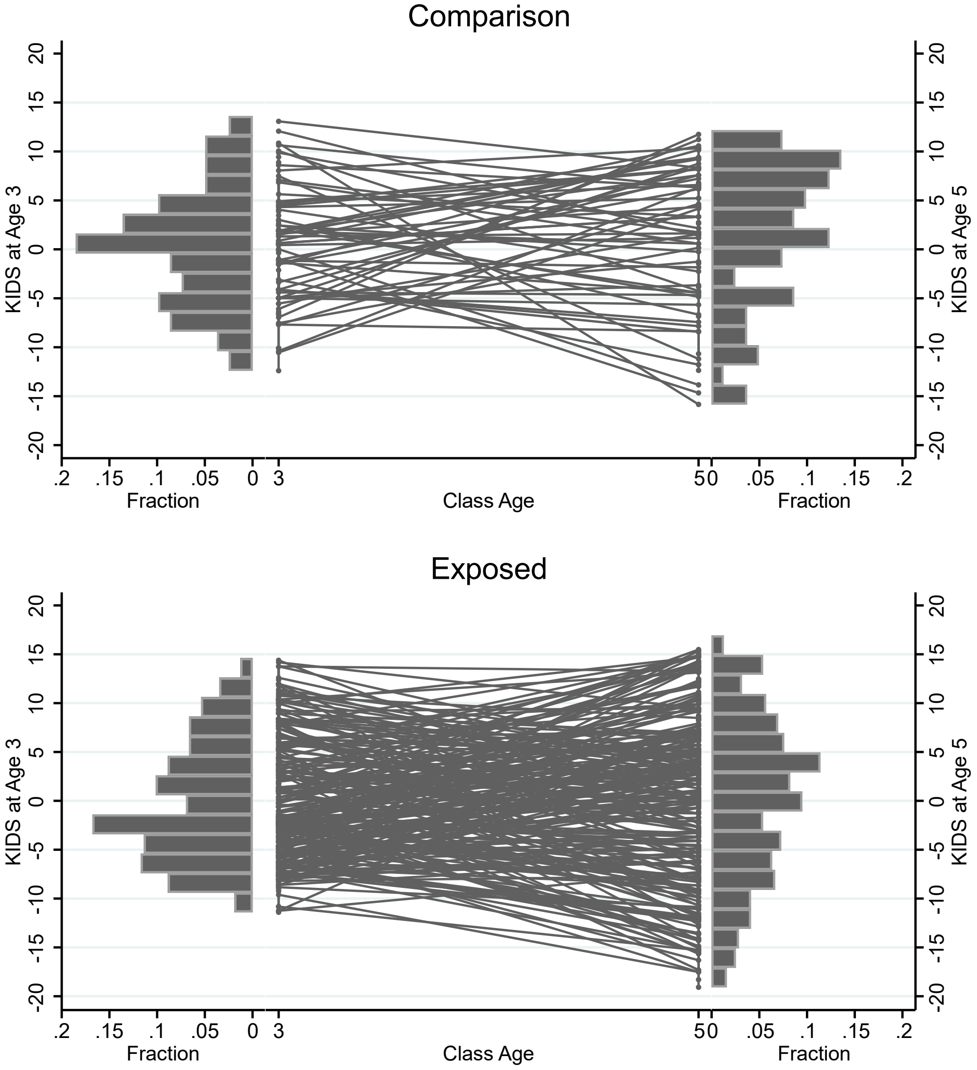

KIDS = the Kinder Infant Development Scale. The KIDS scores were adjusted for age in months, and the score is 0 for typical development.

© 2023 Sato K et al. *JAMA Pediatrics.*

eTable 6. Association Between the Pandemic and Overall Development for 1 to 3 Years Old

Variables	Model 1<br>Coef. (95% CI)	Model 2<br>Coef. (95% CI)	Model 3<br>Coef. (95% CI)	Model 4<br>Coef. (95% CI)	Model 5<br>Coef. (95% CI)
Pandemic	1.04 (-0.44 – 2.63)	1.32 (-0.44 – 3.01)	1.10 (-0.62 – 2.79)	1.03 (-0.71 – 2.73)	0.85 (-0.94 – 2.63)
ITERS		0.11 (-0.45 – 0.66)	-0.17 (-0.74 – 0.41)	0.09 (-0.48 – 0.67)	0.08 (-0.48 – 0.64)
Pandemic x ITERS			2.18 (0.69 – 3.63)		
Depression		0.10 (-0.63 – 0.83)	0.13 (-0.59 – 0.87)	-0.05 (-0.86 – 0.75)	0.13 (-0.61 – 0.86)
Pandemic x Depression				0.84 (-0.90 – 2.60)	
Girl		2.97 (2.29 – 3.66)	2.98 (2.31 – 3.66)	3.00 (2.33 – 3.68)	2.81 (2.06 – 3.56)
Pandemic x Girl					0.87 (-0.65 – 2.40)
Low birth weight		-0.40 (-1.53 – 0.75)	-0.37 (-1.53 – 0.76)	-0.39 (-1.55 – 0.75)	-0.40 (-1.56 – 0.74)
Single parent		0.79 (-2.34 – 4.00)	0.82 (-2.39 – 3.93)	0.79 (-2.42 – 3.89)	0.73 (-2.48 – 3.85)
Sibling		-0.80 (-1.53 – -0.09)	-0.77 (-1.50 – -0.04)	-0.80 (-1.53 – -0.07)	-0.77 (-1.51 – -0.04)
Working mother		-1.14 (-2.94 – 0.65)	-1.17 (-2.90 – 0.56)	-1.22 (-2.97 – 0.52)	-1.20 (-2.95 – 0.53)
Working father		-0.58 (-3.63 – 2.40)	-0.55 (-3.61 – 2.47)	-0.56 (-3.64 – 2.49)	-0.63 (-3.69 – 2.43)
Low income		0.95 (-0.26 – 2.15)	0.97 (-0.22 – 2.15)	0.92 (-0.26 – 2.12)	0.86 (-0.33 – 2.06)
Middle income		0.07 (-0.71 – 0.88)	0.09 (-0.72 – 0.85)	0.08 (-0.73 – 0.84)	0.04 (-0.77 – 0.81)
Days at nursery		0.23 (-0.61 – 1.02)	0.17 (-0.64 – 0.99)	0.19 (-0.63 – 1.01)	0.20 (-0.62 – 1.02)
Random effects					
Level 3: Nursery					
Var(Pandemic)	15.05 (7.81 – 26.42)	15.52 (7.78 – 27.79)	17.93 (9.48 – 31.09)	15.48 (8.14 – 26.66)	15.19 (8.00 – 26.23)
Var(Constant)	0.99 (0.02 – 2.42)	1.03 (0.20 – 2.35)	0.91 (0.16 – 2.18)	1.00 (0.23 – 2.30)	0.98 (0.20 – 2.26)
Level 2: Child					
Var(Pandemic)	18.22 (12.53 – 25.21)	19.04 (12.76 – 26.22)	17.38 (11.73 – 24.21)	18.92 (13.04 – 25.85)	18.89 (13.09 – 25.84)
Var(Constant)	5.67 (3.53 – 7.98)	2.91 (0.30 – 5.18)	3.47 (1.58 – 5.58)	3.37 (1.45 – 5.49)	3.45 (1.54 – 5.57)
Level 1: Time					
Var(Constant)	13.61 (11.51 – 15.99)	13.91 (11.43 – 17.10)	13.46 (11.33 – 15.87)	13.51 (11.40 – 15.93)	13.47 (11.37 – 15.86)

ITERS = the Infant/Toddler Environment Rating Scale, CI = credible interval. All models were also adjusted for child's age and a propensity score.

© 2023 Sato K et al. *JAMA Pediatrics.*

eTable 7. Association Between the Pandemic and Physical Motor for 1 to 3 Years Old

Variables	Model 1<br>Coef. (95% CI)	Model 2<br>Coef. (95% CI)	Model 3<br>Coef. (95% CI)	Model 4<br>Coef. (95% CI)	Model 5<br>Coef. (95% CI)
Pandemic	1.49 (0.13 – 2.95)	1.59 (0.03 – 3.10)	1.50 (-0.05 – 3.03)	1.53 (-0.08 – 3.15)	1.88 (0.16 – 3.59)
ITERS		0.42 (-0.18 – 1.04)	0.28 (-0.35 – 0.93)	0.38 (-0.26 – 1.02)	0.38 (-0.24 – 1.02)
Pandemic x ITERS			0.68 (-0.72 – 2.01)		
Depression		0.03 (-0.75 – 0.79)	0.03 (-0.74 – 0.81)	0.02 (-0.83 – 0.88)	0.03 (-0.75 – 0.81)
Pandemic x Depression				0.06 (-1.82 – 1.95)	
Girl		1.42 (0.70 – 2.14)	1.43 (0.71 – 2.14)	1.43 (0.72 – 2.14)	1.54 (0.76 – 2.33)
Pandemic x Girl					-0.71 (-2.34 – 0.91)
Low birth weight		-0.43 (-1.63 – 0.79)	-0.42 (-1.66 – 0.78)	-0.43 (-1.67 – 0.78)	-0.42 (-1.67 – 0.78)
Single parent		2.25 (-1.02 – 5.60)	2.37 (-1.02 – 5.63)	2.33 (-1.05 – 5.58)	2.37 (-1.00 – 5.62)
Sibling		-0.08 (-0.85 – 0.68)	-0.08 (-0.86 – 0.70)	-0.08 (-0.85 – 0.69)	-0.09 (-0.87 – 0.67)
Working mother		-2.22 (-4.12 – -0.33)	-2.23 (-4.06 – -0.39)	-2.23 (-4.07 – -0.37)	-2.23 (-4.07 – -0.38)
Working father		0.39 (-2.85 – 3.49)	0.49 (-2.74 – 3.74)	0.46 (-2.77 – 3.67)	0.49 (-2.76 – 3.68)
Low income		2.35 (1.07 – 3.65)	2.31 (1.05 – 3.59)	2.29 (1.04 – 3.59)	2.32 (1.07 – 3.61)
Middle income		1.13 (0.27 – 1.99)	1.10 (0.25 – 1.93)	1.09 (0.23 – 1.91)	1.11 (0.24 – 1.94)
Days at nursery		0.23 (-0.64 – 1.05)	0.21 (-0.66 – 1.09)	0.22 (-0.64 – 1.10)	0.24 (-0.63 – 1.12)
Random effects					
Level 3: Nursery					
Var(Pandemic)	8.57 (3.52 – 16.40)	8.67 (3.09 – 17.23)	9.22 (3.66 – 17.50)	8.73 (3.43 – 16.67)	8.87 (3.50 – 16.95)
Var(Constant)	1.55 (0.28 – 3.40)	1.59 (0.35 – 3.43)	1.45 (0.38 – 3.25)	1.49 (0.39 – 3.27)	1.49 (0.39 – 3.31)
Level 2: Child					
Var(Pandemic)	20.26 (13.87 – 27.95)	24.30 (17.05 – 32.67)	23.92 (17.00 – 32.20)	24.19 (17.24 – 32.58)	24.10 (17.23 – 32.48)
Var(Constant)	4.78 (2.29 – 7.50)	2.10 (0.00 – 5.54)	3.31 (1.02 – 5.96)	3.28 (1.01 – 5.92)	3.28 (1.01 – 5.89)
Level 1: Time					
Var(Constant)	15.91 (13.10 – 19.10)	16.58 (12.96 – 20.38)	15.49 (12.68 – 18.53)	15.50 (12.70 – 18.54)	15.50 (12.70 – 18.53)

ITERS = the Infant/Toddler Environment Rating Scale, CI = credible interval. All models were also adjusted for child’s age and a propensity score.

© 2023 Sato K et al. *JAMA Pediatrics.*

eTable 8. Association Between the Pandemic and Manipulation for 1 to 3 Years Old

	Model 1	Model 2	Model 3	Model 4	Model 5
Variables	Coef. (95% CI)	Coef. (95% CI)	Coef. (95% CI)	Coef. (95% CI)	Coef. (95% CI)
Pandemic	2.32 (0.36 – 4.43)	2.54 (0.32 – 4.67)	2.22 (-0.08 – 4.46)	1.86 (-0.36 – 4.03)	1.51 (-0.82 – 3.84)
ITERS		-0.00 (-0.74 – 0.76)	-0.52 (-1.27 – 0.25)	-0.02 (-0.78 – 0.78)	-0.04 (-0.79 – 0.75)
Pandemic x ITERS			4.02 (1.86 – 6.22)		
Depression		-0.13 (-1.13 – 0.85)	-0.09 (-1.05 – 0.89)	-0.55 (-1.63 – 0.54)	-0.09 (-1.06 – 0.87)
Pandemic x Depression				2.41 (-0.09 – 4.99)	
Girl		4.05 (3.18 – 4.93)	4.07 (3.20 – 4.94)	4.10 (3.22 – 4.98)	3.68 (2.70 – 4.65)
Pandemic x Girl					2.15 (-0.05 – 4.36)
Low birth weight		-0.55 (-2.03 – 0.95)	-0.51 (-2.03 – 0.97)	-0.51 (-2.03 – 0.96)	-0.54 (-2.07 – 0.93)
Single parent		0.88 (-3.40 – 5.18)	0.94 (-3.27 – 5.01)	0.90 (-3.32 – 5.03)	0.83 (-3.41 – 4.95)
Sibling		-1.05 (-2.01 – -0.08)	-1.02 (-1.99 – -0.06)	-1.06 (-2.04 – -0.09)	-1.00 (-1.98 – -0.03)
Working mother		-2.12 (-4.53 – 0.28)	-2.13 (-4.44 – 0.17)	-2.23 (-4.56 – 0.06)	-2.18 (-4.50 – 0.14)
Working father		-0.93 (-5.03 – 3.08)	-0.83 (-4.90 – 3.28)	-0.85 (-4.95 – 3.27)	-0.94 (-5.07 – 3.18)
Low income		0.52 (-1.07 – 2.10)	0.57 (-0.98 – 2.10)	0.52 (-1.04 – 2.08)	0.41 (-1.16 – 1.96)
Middle income		0.27 (-0.82 – 1.35)	0.30 (-0.76 – 1.33)	0.30 (-0.77 – 1.33)	0.23 (-0.85 – 1.26)
Days at nursery		0.24 (-0.88 – 1.30)	0.16 (-0.90 – 1.25)	0.17 (-0.90 – 1.29)	0.19 (-0.89 – 1.31)
Random effects					
Level 3: Nursery					
Var(Pandemic)	21.56 (9.77 – 40.27)	20.92 (9.17 – 39.60)	29.04 (13.68 – 52.94)	21.21 (9.60 – 39.35)	19.70 (8.65 – 36.91)
Var(Constant)	2.21 (0.54 – 4.67)	2.07 (0.55 – 4.41)	1.83 (0.52 – 4.03)	2.09 (0.64 – 4.38)	2.04 (0.63 – 4.39)
Level 2: Child					
Var(Pandemic)	45.39 (32.84 – 60.00)	45.39 (32.20 – 60.24)	39.85 (28.30 – 53.85)	44.72 (32.67 – 59.68)	45.12 (33.04 – 59.93)
Var(Constant)	4.72 (1.51 – 8.51)	0.58 (0.00 – 3.36)	0.81 (0.02 – 3.75)	0.77 (0.02 – 3.67)	0.88 (0.03 – 3.98)
Level 1: Time					
Var(Constant)	29.06 (24.74 – 33.95)	29.30 (25.45 – 33.43)	29.09 (25.07 – 33.23)	29.06 (25.09 – 33.07)	28.99 (24.91 – 33.07)

ITERS = the Infant/Toddler Environment Rating Scale, CI = credible interval. All models were also adjusted for child's age and a propensity score.

© 2023 Sato K et al. *JAMA Pediatrics.*

eTable 9. Association Between the Pandemic and Receptive Language for 1 to 3 Years Old

Variables	Model 1<br>Coef. (95% CI)	Model 2<br>Coef. (95% CI)	Model 3<br>Coef. (95% CI)	Model 4<br>Coef. (95% CI)	Model 5<br>Coef. (95% CI)
Pandemic	-0.18 (-1.80 – 1.50)	-0.10 (-1.92 – 1.65)	-0.15 (-1.91 – 1.64)	-0.58 (-2.43 – 1.29)	0.09 (-1.89 – 2.08)
ITERS		0.63 (-0.12 – 1.38)	0.40 (-0.44 – 1.21)	0.64 (-0.14 – 1.42)	0.63 (-0.14 – 1.40)
Pandemic x ITERS			1.00 (-0.44 – 2.45)		
Depression		-0.31 (-1.42 – 0.78)	-0.31 (-1.40 – 0.84)	-0.82 (-2.09 – 0.45)	-0.30 (-1.42 – 0.80)
Pandemic x Depression				1.92 (-0.38 – 4.26)	
Girl		3.03 (1.98 – 4.08)	3.06 (2.03 – 4.10)	3.09 (2.07 – 4.13)	3.15 (1.97 – 4.36)
Pandemic x Girl					-0.42 (-2.43 – 1.57)
Low birth weight		-0.74 (-2.45 – 1.01)	-0.70 (-2.51 – 1.08)	-0.71 (-2.52 – 1.06)	-0.72 (-2.51 – 1.07)
Single parent		2.60 (-2.01 – 7.41)	2.68 (-2.19 – 7.36)	2.57 (-2.26 – 7.27)	2.61 (-2.24 – 7.32)
Sibling		-1.77 (-2.89 – -0.66)	-1.76 (-2.88 – -0.67)	-1.77 (-2.89 – -0.67)	-1.78 (-2.90 – -0.67)
Working mother		-0.99 (-3.63 – 1.62)	-1.06 (-3.67 – 1.51)	-1.07 (-3.69 – 1.49)	-1.05 (-3.68 – 1.52)
Working father		1.40 (-3.10 – 5.86)	1.53 (-3.11 – 6.13)	1.44 (-3.20 – 6.02)	1.46 (-3.17 – 6.07)
Low income		0.02 (-1.71 – 1.81)	0.03 (-1.74 – 1.79)	0.03 (-1.74 – 1.77)	0.03 (-1.74 – 1.79)
Middle income		-0.64 (-1.78 – 0.52)	-0.62 (-1.79 – 0.54)	-0.59 (-1.77 – 0.56)	-0.62 (-1.80 – 0.55)
Days at nursery		-0.26 (-1.52 – 0.95)	-0.30 (-1.54 – 0.95)	-0.33 (-1.57 – 0.92)	-0.27 (-1.51 – 0.98)
Random effects					
Level 3: Nursery					
Var(Pandemic)	5.21 (0.11 – 14.50)	5.91 (0.15 – 15.65)	5.60 (0.00 – 15.92)	5.55 (0.00 – 15.41)	5.12 (0.00 – 14.82)
Var(Constant)	0.58 (0.00 – 2.84)	0.80 (0.00 – 3.03)	0.72 (0.00 – 3.13)	0.79 (0.00 – 3.23)	0.73 (0.00 – 3.16)
Level 2: Child					
Var(Pandemic)	13.51 (2.09 – 26.63)	18.41 (7.93 – 30.94)	17.84 (7.16 – 32.37)	17.94 (7.16 – 32.66)	18.52 (7.90 – 33.08)
Var(Constant)	11.80 (6.86 – 17.01)	9.71 (4.95 – 14.50)	9.78 (4.94 – 14.74)	9.84 (5.00 – 14.77)	9.69 (4.85 – 14.64)
Level 1: Time					
Var(Constant)	35.44 (29.56 – 42.11)	33.35 (27.74 – 39.51)	33.59 (28.18 – 39.77)	33.43 (27.97 – 39.64)	33.63 (28.17 – 39.84)

ITERS = the Infant/Toddler Environment Rating Scale, CI = credible interval. All models were also adjusted for child's age and a propensity score.

© 2023 Sato K et al. *JAMA Pediatrics.*

eTable 10. Association Between the Pandemic and Expressive Language for 1 to 3 Years Old

Variables	Model 1<br>Coef. (95% CI)	Model 2<br>Coef. (95% CI)	Model 3<br>Coef. (95% CI)	Model 4<br>Coef. (95% CI)	Model 5<br>Coef. (95% CI)
Pandemic	-0.23 (-1.70 – 1.28)	-0.24 (-1.88 – 1.36)	-0.29 (-1.90 – 1.36)	-0.43 (-2.10 – 1.28)	-0.51 (-2.30 – 1.31)
ITERS		0.38 (-0.26 – 1.00)	0.23 (-0.44 – 0.87)	0.39 (-0.24 – 1.00)	0.38 (-0.25 – 0.99)
Pandemic x ITERS			0.94 (-0.49 – 2.37)		
Depression		-0.26 (-1.17 – 0.65)	-0.26 (-1.17 – 0.69)	-0.42 (-1.47 – 0.60)	-0.23 (-1.16 – 0.68)
Pandemic x Depression				0.76 (-1.32 – 2.88)	
Girl		3.14 (2.29 – 3.99)	3.14 (2.29 – 3.97)	3.15 (2.31 – 3.99)	2.98 (2.02 – 3.95)
Pandemic x Girl					0.62 (-1.25 – 2.45)
Low birth weight		-1.15 (-2.54 – 0.27)	-1.13 (-2.60 – 0.31)	-1.13 (-2.61 – 0.31)	-1.14 (-2.62 – 0.30)
Single parent		-0.09 (-3.99 – 3.86)	-0.02 (-4.16 – 3.90)	-0.09 (-4.18 – 3.83)	-0.13 (-4.24 – 3.78)
Sibling		-1.09 (-2.02 – -0.18)	-1.05 (-1.98 – -0.12)	-1.06 (-1.98 – -0.13)	-1.04 (-1.96 – -0.11)
Working mother		-0.83 (-3.05 – 1.39)	-0.92 (-3.10 – 1.27)	-0.94 (-3.13 – 1.24)	-0.92 (-3.10 – 1.27)
Working father		-0.71 (-4.57 – 3.06)	-0.65 (-4.53 – 3.23)	-0.70 (-4.58 – 3.15)	-0.76 (-4.63 – 3.10)
Low income		-0.15 (-1.60 – 1.36)	-0.21 (-1.66 – 1.26)	-0.22 (-1.66 – 1.26)	-0.27 (-1.72 – 1.21)
Middle income		-0.45 (-1.41 – 0.55)	-0.46 (-1.47 – 0.52)	-0.45 (-1.46 – 0.53)	-0.49 (-1.50 – 0.51)
Days at nursery		0.31 (-0.73 – 1.33)	0.29 (-0.75 – 1.34)	0.28 (-0.76 – 1.34)	0.29 (-0.74 – 1.35)
Random effects					
Level 3: Nursery					
Var(Pandemic)	5.75 (0.60 – 12.83)	6.63 (1.46 – 14.72)	7.58 (1.82 – 15.76)	6.72 (1.33 – 14.22)	6.94 (1.53 – 14.53)
Var(Constant)	0.19 (0.00 – 1.17)	0.36 (0.00 – 1.62)	0.26 (0.00 – 1.45)	0.28 (0.00 – 1.49)	0.26 (0.00 – 1.43)
Level 2: Child					
Var(Pandemic)	20.87 (12.30 – 30.89)	22.18 (12.44 – 32.75)	20.98 (12.56 – 31.26)	21.58 (12.96 – 31.98)	21.45 (12.97 – 31.80)
Var(Constant)	7.29 (4.02 – 10.63)	3.08 (0.01 – 7.41)	4.68 (1.54 – 7.95)	4.62 (1.49 – 7.91)	4.69 (1.53 – 7.98)
Level 1: Time					
Var(Constant)	24.51 (20.91 – 28.58)	25.85 (21.10 – 30.99)	24.48 (20.83 – 28.62)	24.52 (20.88 – 28.65)	24.49 (20.86 – 28.58)

ITERS = the Infant/Toddler Environment Rating Scale, CI = credible interval. All models were also adjusted for child’s age and a propensity score.

© 2023 Sato K et al. *JAMA Pediatrics.*

eTable 11. Association Between the Pandemic and Language Concepts for 1 to 3 Years Old

Variables	Model 1<br>Coef. (95% CI)	Model 2<br>Coef. (95% CI)	Model 3<br>Coef. (95% CI)	Model 4<br>Coef. (95% CI)	Model 5<br>Coef. (95% CI)
Pandemic	3.30 (1.32 – 5.38)	3.79 (1.56 – 6.00)	3.65 (1.43 – 5.90)	3.72 (1.40 – 6.06)	3.36 (0.83 – 5.95)
ITERS		-0.39 (-1.35 – 0.56)	-0.73 (-1.74 – 0.30)	-0.42 (-1.43 – 0.60)	-0.43 (-1.44 – 0.59)
Pandemic x ITERS			1.88 (-0.13 – 3.88)		
Depression		0.54 (-0.68 – 1.73)	0.56 (-0.63 – 1.76)	0.53 (-0.78 – 1.85)	0.59 (-0.63 – 1.80)
Pandemic x Depression				0.19 (-2.78 – 3.23)	
Girl		2.46 (1.36 – 3.59)	2.50 (1.40 – 3.60)	2.50 (1.39 – 3.59)	2.31 (1.09 – 3.54)
Pandemic x Girl					0.91 (-1.71 – 3.51)
Low birth weight		0.05 (-1.79 – 1.94)	0.04 (-1.88 – 1.90)	0.03 (-1.90 – 1.90)	0.02 (-1.89 – 1.89)
Single parent		-1.23 (-6.47 – 4.11)	-1.33 (-6.64 – 3.90)	-1.43 (-6.75 – 3.76)	-1.48 (-6.81 – 3.73)
Sibling		-1.56 (-2.75 – -0.35)	-1.53 (-2.74 – -0.34)	-1.52 (-2.74 – -0.33)	-1.50 (-2.72 – -0.30)
Working mother		-0.98 (-3.99 – 2.01)	-1.20 (-4.13 – 1.69)	-1.18 (-4.13 – 1.69)	-1.17 (-4.10 – 1.72)
Working father		-2.66 (-7.82 – 2.41)	-2.72 (-7.87 – 2.41)	-2.83 (-8.00 – 2.27)	-2.88 (-8.05 – 2.21)
Low income		1.85 (-0.10 – 3.81)	1.88 (-0.04 – 3.82)	1.81 (-0.12 – 3.76)	1.76 (-0.17 – 3.72)
Middle income		-0.01 (-1.34 – 1.33)	0.01 (-1.32 – 1.30)	-0.02 (-1.35 – 1.26)	-0.05 (-1.37 – 1.24)
Days at nursery		0.26 (-1.12 – 1.57)	0.14 (-1.21 – 1.52)	0.17 (-1.19 – 1.56)	0.17 (-1.20 – 1.56)
Random effects					
Level 3: Nursery					
Var(Pandemic)	10.16 (0.01 – 28.22)	13.46 (1.62 – 33.06)	13.58 (0.50 – 33.91)	13.14 (0.36 – 33.21)	13.45 (0.44 – 33.92)
Var(Constant)	5.97 (2.60 – 11.03)	5.48 (2.30 – 10.29)	5.20 (2.14 – 9.99)	5.37 (2.28 – 10.18)	5.37 (2.28 – 10.20)
Level 2: Child					
Var(Pandemic)	67.80 (49.55 – 91.03)	65.70 (46.55 – 87.21)	65.12 (46.95 – 86.29)	66.41 (48.16 – 87.72)	66.11 (47.96 – 87.25)
Var(Constant)	6.52 (1.76 – 11.66)	3.56 (0.00 – 10.37)	5.76 (0.89 – 11.22)	5.74 (0.88 – 11.21)	5.71 (0.89 – 11.13)
Level 1: Time					
Var(Constant)	40.01 (33.71 – 46.85)	41.58 (33.63 – 49.72)	39.50 (32.83 – 46.90)	39.51 (32.80 – 46.91)	39.54 (32.82 – 46.93)

ITERS = the Infant/Toddler Environment Rating Scale, CI = credible interval. All models were also adjusted for child's age and a propensity score.

© 2023 Sato K et al. *JAMA Pediatrics.*

eTable 12. Association Between the Pandemic and Social Relationships With Children for 1 to 3 Years Old

Variables	Model 1<br>Coef. (95% CI)	Model 2<br>Coef. (95% CI)	Model 3<br>Coef. (95% CI)	Model 4<br>Coef. (95% CI)	Model 5<br>Coef. (95% CI)
Pandemic	2.18 (0.69 – 3.78)	2.35 (0.63 – 4.00)	2.21 (0.49 – 3.86)	2.19 (0.44 – 3.87)	2.06 (0.23 – 3.85)
ITERS		0.21 (-0.38 – 0.80)	-0.04 (-0.66 – 0.58)	0.19 (-0.41 – 0.80)	0.18 (-0.41 – 0.78)
Pandemic x ITERS			1.64 (0.14 – 3.10)		
Depression		0.05 (-0.74 – 0.83)	0.05 (-0.72 – 0.85)	-0.03 (-0.90 – 0.83)	0.07 (-0.71 – 0.85)
Pandemic x Depression				0.44 (-1.45 – 2.38)	
Girl		2.89 (2.17 – 3.62)	2.89 (2.17 – 3.59)	2.90 (2.19 – 3.61)	2.77 (1.96 – 3.58)
Pandemic x Girl					0.55 (-1.11 – 2.22)
Low birth weight		0.36 (-0.85 – 1.59)	0.39 (-0.84 – 1.59)	0.38 (-0.85 – 1.59)	0.38 (-0.86 – 1.59)
Single parent		-0.08 (-3.46 – 3.35)	-0.05 (-3.53 – 3.28)	-0.10 (-3.57 – 3.25)	-0.13 (-3.61 – 3.21)
Sibling		-0.37 (-1.14 – 0.40)	-0.37 (-1.16 – 0.41)	-0.38 (-1.17 – 0.40)	-0.37 (-1.17 – 0.42)
Working mother		-0.20 (-2.13 – 1.73)	-0.30 (-2.18 – 1.55)	-0.31 (-2.19 – 1.55)	-0.30 (-2.17 – 1.57)
Working father		-0.66 (-3.96 – 2.59)	-0.64 (-3.99 – 2.65)	-0.68 (-4.04 – 2.60)	-0.72 (-4.06 – 2.57)
Low income		1.28 (-0.01 – 2.58)	1.28 (0.03 – 2.55)	1.24 (-0.02 – 2.52)	1.20 (-0.06 – 2.49)
Middle income		0.61 (-0.25 – 1.48)	0.61 (-0.26 – 1.44)	0.60 (-0.28 – 1.43)	0.58 (-0.30 – 1.41)
Days at nursery		0.56 (-0.33 – 1.41)	0.51 (-0.36 – 1.41)	0.54 (-0.34 – 1.43)	0.54 (-0.34 – 1.44)
Random effects					
Level 3: Nursery					
Var(Pandemic)	12.54 (5.88 – 23.08)	12.81 (5.77 – 23.83)	14.12 (6.89 – 25.15)	12.73 (6.21 – 22.63)	12.61 (6.18 – 22.43)
Var(Constant)	1.34 (0.23 – 3.00)	1.21 (0.21 – 2.79)	1.12 (0.17 – 2.63)	1.17 (0.23 – 2.66)	1.17 (0.22 – 2.69)
Level 2: Child					
Var(Pandemic)	21.40 (14.71 – 29.55)	21.51 (14.31 – 29.98)	20.53 (13.80 – 28.49)	21.50 (14.72 – 29.64)	21.50 (14.70 – 29.60)
Var(Constant)	4.86 (2.40 – 7.51)	1.44 (0.00 – 4.30)	2.31 (0.27 – 4.69)	2.12 (0.19 – 4.47)	2.21 (0.23 – 4.57)
Level 1: Time					
Var(Constant)	17.08 (14.37 – 20.15)	18.53 (15.27 – 21.86)	17.72 (14.83 – 20.96)	17.88 (14.96 – 21.09)	17.81 (14.94 – 21.02)

ITERS = the Infant/Toddler Environment Rating Scale, CI = credible interval. All models were also adjusted for child's age and a propensity score.

© 2023 Sato K et al. *JAMA Pediatrics.*

eTable 13. Association Between the Pandemic and Social Relationships With Adults for 1 to 3 Years Old

Variables	Model 1<br>Coef. (95% CI)	Model 2<br>Coef. (95% CI)	Model 3<br>Coef. (95% CI)	Model 4<br>Coef. (95% CI)	Model 5<br>Coef. (95% CI)
Pandemic	2.66 (0.19 – 5.31)	3.00 (0.16 – 5.82)	2.75 (-0.03 – 5.50)	3.03 (0.18 – 5.77)	2.22 (-0.78 – 5.16)
ITERS		0.02 (-0.89 – 0.93)	-0.22 (-1.15 – 0.74)	-0.02 (-0.96 – 0.95)	-0.02 (-0.96 – 0.94)
Pandemic x ITERS			1.76 (-0.80 – 4.35)		
Depression		0.01 (-1.13 – 1.15)	0.01 (-1.11 – 1.15)	0.11 (-1.14 – 1.37)	0.05 (-1.09 – 1.18)
Pandemic x Depression				-0.64 (-3.71 – 2.55)	
Girl		3.53 (2.51 – 4.57)	3.55 (2.52 – 4.57)	3.55 (2.52 – 4.57)	3.31 (2.18 – 4.42)
Pandemic x Girl					1.47 (-1.25 – 4.17)
Low birth weight		0.52 (-1.22 – 2.26)	0.55 (-1.23 – 2.28)	0.53 (-1.25 – 2.28)	0.53 (-1.25 – 2.27)
Single parent		3.05 (-1.90 – 8.08)	3.12 (-1.78 – 7.97)	3.07 (-1.85 – 7.91)	3.03 (-1.90 – 7.87)
Sibling		-0.80 (-1.95 – 0.36)	-0.79 (-1.95 – 0.35)	-0.79 (-1.94 – 0.36)	-0.77 (-1.92 – 0.38)
Working mother		-1.03 (-3.82 – 1.79)	-1.11 (-3.90 – 1.62)	-1.11 (-3.90 – 1.60)	-1.11 (-3.91 – 1.61)
Working father		1.64 (-3.18 – 6.36)	1.77 (-3.03 – 6.69)	1.71 (-3.11 – 6.59)	1.68 (-3.14 – 6.58)
Low income		0.74 (-1.13 – 2.60)	0.77 (-1.06 – 2.60)	0.71 (-1.12 – 2.56)	0.66 (-1.18 – 2.50)
Middle income		-0.36 (-1.65 – 0.92)	-0.32 (-1.57 – 0.88)	-0.36 (-1.60 – 0.86)	-0.38 (-1.64 – 0.85)
Days at nursery		-0.42 (-1.73 – 0.82)	-0.46 (-1.71 – 0.84)	-0.43 (-1.68 – 0.89)	-0.45 (-1.71 – 0.86)
Random effects					
Level 3: Nursery					
Var(Pandemic)	39.41 (19.12 – 71.15)	39.01 (18.20 – 71.09)	43.41 (21.30 – 77.66)	38.64 (19.18 – 70.11)	38.09 (18.82 – 68.56)
Var(Constant)	4.45 (1.69 – 8.70)	4.61 (1.84 – 8.97)	4.49 (1.81 – 8.68)	4.52 (1.85 – 8.71)	4.54 (1.85 – 8.76)
Level 2: Child					
Var(Pandemic)	76.98 (57.94 – 99.65)	76.62 (57.64 – 98.64)	74.82 (57.43 – 97.18)	76.67 (58.76 – 99.27)	76.61 (58.78 – 98.89)
Var(Constant)	1.97 (0.12 – 5.49)	0.44 (0.00 – 2.76)	0.66 (0.01 – 3.83)	0.65 (0.01 – 3.76)	0.67 (0.01 – 3.88)
Level 1: Time					
Var(Constant)	40.54 (35.03 – 46.47)	39.40 (34.49 – 44.71)	39.24 (34.25 – 44.69)	39.26 (34.30 – 44.71)	39.22 (34.25 – 44.66)

ITERS = the Infant/Toddler Environment Rating Scale, CI = credible interval. All models were also adjusted for child’s age and a propensity score.

© 2023 Sato K et al. *JAMA Pediatrics.*

eTable 14. Association Between the Pandemic and Discipline for 1 to 3 Years Old

Variables	Model 1<br>Coef. (95% CI)	Model 2<br>Coef. (95% CI)	Model 3<br>Coef. (95% CI)	Model 4<br>Coef. (95% CI)	Model 5<br>Coef. (95% CI)
Pandemic	-1.29 (-3.38 – 0.94)	-1.05 (-3.47 – 1.36)	-1.35 (-3.72 – 0.99)	-1.48 (-3.81 – 0.85)	-1.00 (-3.44 – 1.40)
ITERS		-0.21 (-0.86 – 0.44)	-0.54 (-1.21 – 0.16)	-0.23 (-0.90 – 0.46)	-0.23 (-0.90 – 0.45)
Pandemic x ITERS			2.57 (0.63 – 4.44)		
Depression		0.60 (-0.29 – 1.46)	0.60 (-0.25 – 1.48)	0.39 (-0.59 – 1.37)	0.60 (-0.27 – 1.47)
Pandemic x Depression				1.07 (-1.12 – 3.31)	
Girl		3.00 (2.22 – 3.80)	3.00 (2.22 – 3.78)	3.03 (2.25 – 3.81)	3.09 (2.20 – 3.95)
Pandemic x Girl					-0.45 (-2.35 – 1.50)
Low birth weight		-0.59 (-1.92 – 0.76)	-0.57 (-1.92 – 0.77)	-0.57 (-1.92 – 0.77)	-0.58 (-1.92 – 0.76)
Single parent		2.31 (-1.48 – 6.12)	2.37 (-1.39 – 6.05)	2.31 (-1.47 – 5.99)	2.32 (-1.47 – 6.01)
Sibling		-0.14 (-1.01 – 0.73)	-0.11 (-0.98 – 0.75)	-0.14 (-1.01 – 0.72)	-0.14 (-1.01 – 0.72)
Working mother		-0.82 (-2.95 – 1.34)	-0.86 (-2.97 – 1.18)	-0.90 (-3.02 – 1.16)	-0.89 (-3.00 – 1.19)
Working father		1.55 (-2.11 – 5.13)	1.65 (-2.03 – 5.34)	1.60 (-2.05 – 5.31)	1.60 (-2.07 – 5.31)
Low income		0.78 (-0.63 – 2.19)	0.85 (-0.54 – 2.21)	0.78 (-0.60 – 2.16)	0.79 (-0.59 – 2.17)
Middle income		0.17 (-0.79 – 1.12)	0.21 (-0.75 – 1.12)	0.19 (-0.77 – 1.11)	0.18 (-0.78 – 1.10)
Days at nursery		0.31 (-0.67 – 1.26)	0.24 (-0.72 – 1.23)	0.27 (-0.69 – 1.28)	0.30 (-0.67 – 1.30)
Random effects					
Level 3: Nursery					
Var(Pandemic)	35.80 (20.08 – 59.97)	35.79 (19.63 – 61.23)	39.58 (22.20 – 65.15)	35.66 (20.09 – 58.55)	35.71 (20.09 – 58.46)
Var(Constant)	1.41 (0.01 – 3.32)	1.45 (0.20 – 3.40)	1.38 (0.26 – 3.16)	1.44 (0.30 – 3.23)	1.43 (0.31 – 3.28)
Level 2: Child					
Var(Pandemic)	27.40 (18.88 – 37.72)	29.52 (20.15 – 39.97)	27.66 (19.13 – 38.12)	29.44 (20.70 – 40.15)	29.51 (20.83 – 40.23)
Var(Constant)	3.61 (1.21 – 6.33)	0.56 (0.00 – 3.21)	0.85 (0.02 – 3.47)	0.80 (0.02 – 3.32)	0.78 (0.02 – 3.31)
Level 1: Time					
Var(Constant)	23.56 (20.06 – 27.47)	24.02 (20.60 – 27.53)	23.75 (20.31 – 27.23)	23.79 (20.44 – 27.25)	23.83 (20.48 – 27.25)

ITERS = the Infant/Toddler Environment Rating Scale, CI = credible interval. All models were also adjusted for child's age and a propensity score.

© 2023 Sato K et al. *JAMA Pediatrics.*

eTable 15. Association Between the Pandemic and Overall Development for 3 to 5 Years Old

Variables	Model 1<br>Coef. (95% CI)	Model 2<br>Coef. (95% CI)	Model 3<br>Coef. (95% CI)	Model 4<br>Coef. (95% CI)	Model 5<br>Coef. (95% CI)
Pandemic	-3.81 (-7.19 – -0.58)	-4.39 (-7.66 – -1.27)	-4.69 (-7.92 – -1.56)	-3.86 (-6.99 – -0.69)	-4.63 (-7.84 – -1.45)
ECERS		0.96 (-0.22 – 2.15)	1.33 (0.07 – 2.61)	0.98 (-0.21 – 2.20)	1.09 (-0.09 – 2.30)
Pandemic x ECERS			-1.35 (-3.30 – 0.67)		
Depression		-0.15 (-1.30 – 0.98)	-0.18 (-1.31 – 0.92)	0.65 (-0.65 – 1.95)	-0.14 (-1.28 – 0.99)
Pandemic x Depression				-2.62 (-4.80 – -0.49)	
Girl		2.52 (1.40 – 3.63)	2.48 (1.43 – 3.55)	2.52 (1.47 – 3.60)	2.56 (1.40 – 3.77)
Pandemic x Girl					-0.28 (-2.04 – 1.39)
Low birth weight		-1.79 (-3.62 – 0.05)	-1.84 (-3.60 – -0.08)	-1.76 (-3.52 – 0.00)	-1.81 (-3.58 – -0.04)
Single parent		1.16 (-5.08 – 7.34)	1.27 (-5.20 – 7.66)	0.93 (-5.57 – 7.34)	1.30 (-5.16 – 7.70)
Sibling		1.20 (-0.02 – 2.38)	1.21 (-0.02 – 2.41)	1.13 (-0.10 – 2.32)	1.22 (-0.01 – 2.41)
Working mother		-2.50 (-5.32 – 0.53)	-2.36 (-5.24 – 0.62)	-2.63 (-5.49 – 0.40)	-2.49 (-5.35 – 0.53)
Working father		0.83 (-5.17 – 6.78)	0.89 (-5.35 – 7.20)	0.50 (-5.64 – 6.79)	0.97 (-5.23 – 7.32)
Low income		-2.14 (-3.86 – -0.44)	-2.19 (-3.79 – -0.54)	-2.10 (-3.69 – -0.45)	-2.19 (-3.76 – -0.52)
Middle income		-0.76 (-1.92 – 0.43)	-0.79 (-1.99 – 0.39)	-0.73 (-1.93 – 0.44)	-0.80 (-2.00 – 0.39)
Days at nursery		0.80 (-0.65 – 2.26)	0.78 (-0.67 – 2.29)	0.74 (-0.72 – 2.24)	0.79 (-0.67 – 2.29)
Random effects					
Level 3: Nursery					
Var(Pandemic)	32.08 (13.79 – 67.15)	32.21 (13.98 – 66.67)	34.18 (14.62 – 72.40)	31.82 (13.73 – 68.12)	64.32 (28.20 – 134.81)
Var(Constant)	17.30 (6.95 – 38.05)	17.83 (7.30 – 37.43)	18.12 (7.28 – 38.75)	17.68 (7.10 – 38.36)	31.90 (13.61 – 68.85)
Level 2: Child					
Var(Pandemic)	18.40 (8.99 – 28.50)	20.13 (11.69 – 30.11)	19.53 (10.80 – 29.37)	19.42 (10.84 – 29.08)	17.87 (7.17 – 38.53)
Var(Constant)	20.18 (15.46 – 25.30)	17.85 (13.14 – 22.85)	17.98 (13.16 – 23.06)	17.80 (13.05 – 22.88)	20.02 (11.35 – 29.90)
Level 1: Time					
Var(Constant)	24.40 (20.02 – 29.11)	23.80 (19.58 – 28.52)	23.69 (19.53 – 28.48)	23.71 (19.57 – 28.47)	17.93 (13.09 – 23.03)

ECERS = the Early Childhood Environment Rating Scale, CI = credible interval. All models were also adjusted for child's age and a propensity score.

© 2023 Sato K et al. *JAMA Pediatrics.*

eTable 16. Association Between the Pandemic and Physical Motor for 3 to 5 Years Old

Variables	Model 1<br>Coef. (95% CI)	Model 2<br>Coef. (95% CI)	Model 3<br>Coef. (95% CI)	Model 4<br>Coef. (95% CI)	Model 5<br>Coef. (95% CI)
Pandemic	-4.08 (-7.45 – -0.64)	-4.99 (-8.95 – -1.52)	-5.15 (-8.56 – -1.87)	-4.52 (-7.99 – -1.19)	-5.08 (-8.62 – -1.66)
ECERS		1.17 (0.06 – 2.27)	1.36 (0.18 – 2.54)	1.20 (0.08 – 2.31)	1.26 (0.15 – 2.37)
Pandemic x ECERS			-0.71 (-3.17 – 1.86)		
Depression		-0.95 (-2.17 – 0.28)	-0.94 (-2.16 – 0.27)	-0.39 (-1.79 – 1.01)	-0.93 (-2.17 – 0.31)
Pandemic x Depression				-2.27 (-5.03 – 0.42)	
Girl		0.48 (-0.56 – 1.54)	0.45 (-0.58 – 1.50)	0.48 (-0.55 – 1.52)	0.52 (-0.65 – 1.69)
Pandemic x Girl					-0.25 (-2.60 – 1.97)
Low birth weight		-1.18 (-3.03 – 0.73)	-1.22 (-3.07 – 0.61)	-1.16 (-3.00 – 0.69)	-1.21 (-3.04 – 0.65)
Single parent		1.72 (-5.36 – 8.76)	1.80 (-5.39 – 9.09)	1.58 (-5.60 – 8.85)	1.82 (-5.35 – 9.16)
Sibling		2.48 (1.24 – 3.68)	2.48 (1.25 – 3.71)	2.44 (1.20 – 3.66)	2.48 (1.24 – 3.70)
Working mother		1.02 (-2.13 – 4.24)	1.09 (-2.09 – 4.27)	0.89 (-2.31 – 4.06)	1.03 (-2.16 – 4.23)
Working father		0.08 (-6.87 – 6.94)	0.13 (-6.67 – 7.33)	-0.09 (-6.90 – 7.08)	0.17 (-6.58 – 7.38)
Low income		-1.01 (-2.75 – 0.73)	-1.06 (-2.70 – 0.70)	-1.00 (-2.63 – 0.74)	-1.05 (-2.68 – 0.69)
Middle income		0.39 (-0.80 – 1.63)	0.37 (-0.84 – 1.56)	0.41 (-0.80 – 1.61)	0.37 (-0.83 – 1.57)
Days at nursery		1.39 (-0.14 – 2.92)	1.39 (-0.13 – 2.94)	1.36 (-0.17 – 2.91)	1.39 (-0.13 – 2.94)
Random effects					
Level 3: Nursery					
Var(Pandemic)	33.58 (13.43 – 72.22)	34.87 (14.04 – 79.46)	33.38 (13.23 – 73.64)	34.07 (13.70 – 75.07)	64.32 (28.20 – 134.81)
Var(Constant)	9.24 (3.44 – 20.92)	8.48 (2.73 – 19.45)	7.74 (2.70 – 17.53)	7.71 (2.66 – 17.82)	33.38 (13.26 – 73.97)
Level 2: Child					
Var(Pandemic)	48.26 (34.04 – 63.60)	47.94 (34.21 – 63.39)	48.10 (33.65 – 65.04)	47.70 (33.38 – 64.23)	7.72 (2.66 – 17.86)
Var(Constant)	7.69 (3.22 – 12.80)	5.41 (0.57 – 10.64)	5.49 (0.50 – 11.56)	5.56 (0.52 – 11.58)	48.18 (33.66 – 64.75)
Level 1: Time					
Var(Constant)	37.03 (31.09 – 43.56)	38.18 (31.81 – 45.42)	37.93 (31.65 – 44.93)	37.79 (31.55 – 44.73)	5.58 (0.53 – 11.63)

ECERS = the Early Childhood Environment Rating Scale, CI = credible interval. All models were also adjusted for child’s age and a propensity score.

© 2023 Sato K et al. *JAMA Pediatrics.*

eTable 17. Association Between the Pandemic and Manipulation for 3 to 5 Years Old

Variables	Model 1<br>Coef. (95% CI)	Model 2<br>Coef. (95% CI)	Model 3<br>Coef. (95% CI)	Model 4<br>Coef. (95% CI)	Model 5<br>Coef. (95% CI)
Pandemic	-3.55 (-7.48 – 0.21)	-3.79 (-8.37 – -0.07)	-4.04 (-7.73 – -0.46)	-2.95 (-6.51 – 0.68)	-3.75 (-7.42 – -0.10)
ECERS		0.43 (-0.97 – 1.83)	0.83 (-0.69 – 2.36)	0.44 (-0.96 – 1.89)	0.59 (-0.81 – 2.05)
Pandemic x ECERS			-1.36 (-3.54 – 0.91)		
Depression		-0.00 (-1.32 – 1.30)	-0.02 (-1.32 – 1.23)	1.01 (-0.49 – 2.48)	0.03 (-1.28 – 1.32)
Pandemic x Depression				-3.29 (-5.76 – -0.88)	
Girl		4.36 (3.05 – 5.66)	4.31 (3.08 – 5.57)	4.36 (3.13 – 5.62)	4.52 (3.17 – 5.93)
Pandemic x Girl					-0.74 (-2.70 – 1.15)
Low birth weight		-2.17 (-4.30 – -0.04)	-2.23 (-4.29 – -0.19)	-2.13 (-4.17 – -0.08)	-2.20 (-4.25 – -0.16)
Single parent		0.15 (-7.04 – 7.17)	0.28 (-7.18 – 7.75)	-0.18 (-7.63 – 7.29)	0.25 (-7.17 – 7.73)
Sibling		0.52 (-0.90 – 1.89)	0.53 (-0.91 – 1.93)	0.43 (-1.01 – 1.82)	0.54 (-0.90 – 1.93)
Working mother		-2.24 (-5.51 – 1.20)	-2.14 (-5.43 – 1.28)	-2.40 (-5.69 – 1.00)	-2.27 (-5.56 – 1.14)
Working father		-0.07 (-7.01 – 6.78)	-0.01 (-7.10 – 7.27)	-0.55 (-7.58 – 6.75)	0.03 (-7.05 – 7.39)
Low income		-1.82 (-3.83 – 0.16)	-1.88 (-3.69 – 0.05)	-1.76 (-3.57 – 0.17)	-1.87 (-3.69 – 0.05)
Middle income		-0.68 (-2.02 – 0.71)	-0.71 (-2.14 – 0.67)	-0.63 (-2.05 – 0.75)	-0.72 (-2.15 – 0.67)
Days at nursery		1.46 (-0.20 – 3.16)	1.45 (-0.21 – 3.17)	1.40 (-0.26 – 3.12)	1.45 (-0.21 – 3.18)
Random effects					
Level 3: Nursery					
Var(Pandemic)	42.49 (17.91 – 89.79)	43.08 (18.24 – 92.26)	44.28 (18.94 – 94.71)	41.31 (17.55 – 88.31)	64.32 (28.20 – 134.81)
Var(Constant)	36.49 (15.48 – 78.38)	39.93 (16.37 – 102.79)	35.88 (15.40 – 75.91)	35.64 (15.36 – 75.97)	41.71 (17.38 – 89.74)
Level 2: Child					
Var(Pandemic)	23.75 (9.06 – 38.38)	26.75 (15.12 – 40.63)	26.65 (15.70 – 39.73)	25.77 (14.70 – 38.47)	35.67 (15.34 – 75.67)
Var(Constant)	31.55 (24.70 – 38.95)	27.08 (20.55 – 34.21)	27.58 (20.75 – 34.79)	27.03 (20.25 – 34.15)	26.86 (15.60 – 40.02)
Level 1: Time					
Var(Constant)	30.30 (24.21 – 37.46)	29.11 (23.25 – 35.58)	28.61 (22.95 – 35.00)	28.90 (23.23 – 35.43)	27.25 (20.39 – 34.39)

ECERS = the Early Childhood Environment Rating Scale, CI = credible interval. All models were also adjusted for child’s age and a propensity score.

© 2023 Sato K et al. *JAMA Pediatrics.*

eTable 18. Association Between the Pandemic and Receptive Language for 3 to 5 Years Old

Variables	Model 1<br>Coef. (95% CI)	Model 2<br>Coef. (95% CI)	Model 3<br>Coef. (95% CI)	Model 4<br>Coef. (95% CI)	Model 5<br>Coef. (95% CI)
Pandemic	-2.36 (-4.93 – 0.31)	-3.11 (-5.89 – -0.35)	-3.25 (-6.12 – -0.52)	-2.98 (-5.89 – -0.17)	-2.91 (-5.91 – -0.06)
ECERS		0.46 (-0.96 – 1.86)	0.96 (-0.57 – 2.51)	0.54 (-0.88 – 2.01)	0.58 (-0.84 – 2.04)
Pandemic x ECERS			-1.59 (-3.72 – 0.54)		
Depression		-0.66 (-2.03 – 0.74)	-0.70 (-2.07 – 0.65)	-0.35 (-2.02 – 1.29)	-0.64 (-2.03 – 0.74)
Pandemic x Depression				-0.84 (-3.42 – 1.74)	
Girl		2.79 (1.45 – 4.12)	2.75 (1.50 – 4.03)	2.76 (1.50 – 4.04)	3.02 (1.55 – 4.49)
Pandemic x Girl					-0.75 (-2.87 – 1.24)
Low birth weight		-1.70 (-3.92 – 0.52)	-1.78 (-3.89 – 0.37)	-1.72 (-3.85 – 0.46)	-1.73 (-3.85 – 0.44)
Single parent		3.51 (-3.81 – 10.91)	3.58 (-3.96 – 11.02)	3.54 (-4.01 – 10.99)	3.57 (-3.99 – 11.03)
Sibling		0.58 (-0.89 – 1.98)	0.61 (-0.86 – 2.04)	0.59 (-0.91 – 2.02)	0.61 (-0.87 – 2.05)
Working mother		-1.37 (-4.89 – 2.33)	-1.14 (-4.73 – 2.48)	-1.36 (-4.94 – 2.27)	-1.36 (-4.92 – 2.27)
Working father		1.68 (-5.44 – 8.72)	1.71 (-5.64 – 8.99)	1.70 (-5.68 – 9.10)	1.76 (-5.62 – 9.13)
Low income		-4.13 (-6.16 – -2.14)	-4.18 (-6.12 – -2.16)	-4.15 (-6.09 – -2.14)	-4.17 (-6.12 – -2.15)
Middle income		-1.44 (-2.83 – -0.00)	-1.46 (-2.88 – -0.09)	-1.46 (-2.89 – -0.08)	-1.47 (-2.91 – -0.09)
Days at nursery		1.01 (-0.71 – 2.77)	0.97 (-0.80 – 2.81)	0.98 (-0.80 – 2.81)	0.99 (-0.79 – 2.83)
Random effects					
Level 3: Nursery					
Var(Pandemic)	12.58 (4.01 – 30.35)	12.73 (4.03 – 29.97)	13.82 (4.08 – 33.79)	12.23 (3.43 – 29.94)	64.32 (28.20 – 134.81)
Var(Constant)	20.06 (7.67 – 45.27)	20.66 (8.12 – 45.07)	20.72 (8.11 – 45.69)	20.71 (8.06 – 46.20)	12.20 (3.36 – 29.98)
Level 2: Child					
Var(Pandemic)	10.80 (0.17 – 27.16)	11.42 (0.84 – 26.70)	8.66 (0.86 – 21.93)	9.48 (0.98 – 23.05)	20.46 (8.03 – 44.97)
Var(Constant)	26.05 (19.32 – 33.64)	22.25 (15.29 – 29.48)	22.34 (15.06 – 30.06)	22.13 (14.87 – 29.83)	9.52 (0.91 – 23.26)
Level 1: Time					
Var(Constant)	43.24 (35.21 – 51.54)	43.31 (35.21 – 52.00)	43.90 (36.53 – 52.34)	43.98 (36.49 – 52.60)	22.24 (14.95 – 29.91)

ECERS = the Early Childhood Environment Rating Scale, CI = credible interval. All models were also adjusted for child's age and a propensity score.

© 2023 Sato K et al. *JAMA Pediatrics.*

eTable 19. Association Between the Pandemic and Expressive Language for 3 to 5 Years Old

Variables	Model 1<br>Coef. (95% CI)	Model 2<br>Coef. (95% CI)	Model 3<br>Coef. (95% CI)	Model 4<br>Coef. (95% CI)	Model 5<br>Coef. (95% CI)
Pandemic	-4.99 (-7.20 – -2.69)	-5.64 (-8.07 – -3.19)	-5.73 (-8.20 – -3.35)	-5.30 (-7.87 – -2.81)	-6.55 (-9.23 – -3.99)
ECERS		0.89 (-0.53 – 2.31)	1.15 (-0.37 – 2.72)	0.97 (-0.47 – 2.46)	1.03 (-0.40 – 2.51)
Pandemic x ECERS			-0.63 (-2.59 – 1.31)		
Depression		-0.08 (-1.54 – 1.36)	-0.09 (-1.54 – 1.30)	0.35 (-1.33 – 2.02)	-0.13 (-1.58 – 1.32)
Pandemic x Depression				-1.40 (-4.23 – 1.37)	
Girl		3.19 (1.78 – 4.59)	3.15 (1.81 – 4.51)	3.17 (1.83 – 4.55)	2.70 (1.22 – 4.23)
Pandemic x Girl					1.63 (-0.65 – 3.77)
Low birth weight		-1.83 (-4.14 – 0.49)	-1.89 (-4.14 – 0.37)	-1.84 (-4.09 – 0.41)	-1.90 (-4.14 – 0.38)
Single parent		2.06 (-5.93 – 9.93)	2.12 (-6.03 – 10.34)	1.93 (-6.30 – 10.12)	2.37 (-5.80 – 10.58)
Sibling		-0.09 (-1.65 – 1.39)	-0.08 (-1.62 – 1.42)	-0.13 (-1.69 – 1.38)	-0.07 (-1.62 – 1.41)
Working mother		-1.75 (-5.37 – 2.07)	-1.67 (-5.35 – 2.11)	-1.82 (-5.50 – 1.99)	-1.58 (-5.24 – 2.23)
Working father		0.58 (-7.10 – 8.22)	0.61 (-7.27 – 8.63)	0.40 (-7.59 – 8.44)	0.86 (-7.05 – 8.91)
Low income		-3.03 (-5.20 – -0.88)	-3.07 (-5.11 – -0.95)	-3.03 (-5.05 – -0.91)	-3.07 (-5.08 – -0.93)
Middle income		-0.73 (-2.21 – 0.79)	-0.77 (-2.30 – 0.70)	-0.75 (-2.26 – 0.73)	-0.80 (-2.33 – 0.67)
Days at nursery		0.04 (-1.79 – 1.90)	0.02 (-1.82 – 1.94)	0.02 (-1.84 – 1.95)	0.03 (-1.82 – 1.95)
Random effects					
Level 3: Nursery					
Var(Pandemic)	3.39 (0.00 – 14.40)	4.00 (0.01 – 14.58)	3.45 (0.00 – 15.25)	2.92 (0.00 – 13.09)	64.32 (28.20 – 134.81)
Var(Constant)	13.94 (5.07 – 32.15)	13.57 (5.20 – 30.01)	13.80 (5.01 – 30.71)	13.83 (5.03 – 30.99)	2.85 (0.00 – 13.05)
Level 2: Child					
Var(Pandemic)	34.45 (17.94 – 51.39)	32.51 (18.28 – 49.46)	32.52 (18.74 – 48.69)	32.61 (18.83 – 48.65)	13.78 (5.10 – 30.81)
Var(Constant)	29.97 (22.47 – 38.08)	26.80 (19.27 – 34.85)	26.86 (18.98 – 34.80)	26.78 (18.95 – 34.69)	32.53 (18.78 – 48.44)
Level 1: Time					
Var(Constant)	40.04 (33.04 – 47.62)	40.71 (33.47 – 48.77)	40.64 (33.65 – 48.63)	40.68 (33.72 – 48.53)	27.02 (19.11 – 34.94)

ECERS = the Early Childhood Environment Rating Scale, CI = credible interval. All models were also adjusted for child’s age and a propensity score.

© 2023 Sato K et al. *JAMA Pediatrics.*

eTable 20. Association Between the Pandemic and Language Concepts for 3 to 5 Years Old

Variables	Model 1<br>Coef. (95% CI)	Model 2<br>Coef. (95% CI)	Model 3<br>Coef. (95% CI)	Model 4<br>Coef. (95% CI)	Model 5<br>Coef. (95% CI)
Pandemic	-1.42 (-4.91 – 2.15)	-1.99 (-5.85 – 1.79)	-2.32 (-6.39 – 1.56)	-1.57 (-5.45 – 2.19)	-1.84 (-5.85 – 1.99)
ECERS		0.38 (-1.28 – 2.01)	1.39 (-0.54 – 3.30)	0.42 (-1.30 – 2.14)	0.50 (-1.21 – 2.21)
Pandemic x ECERS			-3.24 (-6.30 – -0.27)		
Depression		-0.32 (-2.01 – 1.43)	-0.38 (-2.06 – 1.31)	0.45 (-1.70 – 2.62)	-0.30 (-2.03 – 1.45)
Pandemic x Depression				-1.92 (-5.15 – 1.33)	
Girl		1.63 (0.05 – 3.19)	1.61 (0.12 – 3.12)	1.63 (0.13 – 3.15)	1.91 (0.07 – 3.77)
Pandemic x Girl					-0.78 (-3.48 – 1.84)
Low birth weight		-2.78 (-5.41 – -0.05)	-2.88 (-5.45 – -0.30)	-2.76 (-5.35 – -0.15)	-2.80 (-5.41 – -0.21)
Single parent		0.13 (-8.86 – 9.25)	0.16 (-9.15 – 9.35)	0.03 (-9.26 – 9.25)	0.23 (-9.09 – 9.41)
Sibling		1.74 (-0.02 – 3.45)	1.76 (-0.01 – 3.51)	1.70 (-0.08 – 3.45)	1.76 (-0.03 – 3.50)
Working mother		-3.64 (-8.17 – 1.02)	-3.32 (-7.80 – 1.23)	-3.77 (-8.32 – 0.80)	-3.65 (-8.20 – 0.90)
Working father		0.56 (-8.32 – 9.49)	0.44 (-8.64 – 9.34)	0.41 (-8.88 – 9.39)	0.65 (-8.56 – 9.64)
Low income		-2.45 (-4.91 – -0.03)	-2.54 (-4.92 – -0.07)	-2.44 (-4.82 – 0.04)	-2.49 (-4.86 – -0.00)
Middle income		-1.25 (-2.94 – 0.45)	-1.27 (-2.96 – 0.42)	-1.24 (-2.92 – 0.47)	-1.27 (-2.96 – 0.43)
Days at nursery		-0.32 (-2.49 – 1.84)	-0.30 (-2.42 – 1.89)	-0.36 (-2.51 – 1.82)	-0.33 (-2.47 – 1.86)
Random effects					
Level 3: Nursery					
Var(Pandemic)	27.72 (8.60 – 66.81)	28.41 (8.55 – 66.72)	39.08 (12.51 – 93.87)	26.76 (7.57 – 66.54)	64.32 (28.20 – 134.81)
Var(Constant)	23.16 (8.35 – 53.36)	25.21 (9.51 – 56.47)	27.44 (10.07 – 60.89)	25.28 (9.18 – 56.13)	27.35 (7.58 – 68.23)
Level 2: Child					
Var(Pandemic)	1.43 (0.00 – 6.45)	0.52 (0.00 – 2.71)	0.24 (0.00 – 2.86)	0.26 (0.00 – 3.09)	25.12 (9.13 – 55.86)
Var(Constant)	18.92 (8.91 – 30.24)	19.64 (8.42 – 30.44)	19.82 (9.29 – 31.01)	19.52 (8.93 – 30.78)	0.27 (0.00 – 3.17)
Level 1: Time					
Var(Constant)	89.47 (77.43 – 102.78)	87.70 (75.83 – 101.36)	86.31 (74.86 – 99.44)	87.70 (76.16 – 101.08)	19.52 (8.83 – 30.81)

ECERS = the Early Childhood Environment Rating Scale, CI = credible interval. All models were also adjusted for child's age and a propensity score.

© 2023 Sato K et al. *JAMA Pediatrics.*

eTable 21. Association Between the Pandemic and Social Relationships With Children for 3 to 5 Years Old

Variables	Model 1<br>Coef. (95% CI)	Model 2<br>Coef. (95% CI)	Model 3<br>Coef. (95% CI)	Model 4<br>Coef. (95% CI)	Model 5<br>Coef. (95% CI)
Pandemic	-1.66 (-4.31 – 1.03)	-1.92 (-4.69 – 0.76)	-2.17 (-4.91 – 0.44)	-1.02 (-3.77 – 1.67)	-1.85 (-4.64 – 0.82)
ECERS		0.64 (-0.53 – 1.80)	0.83 (-0.46 – 2.12)	0.60 (-0.57 – 1.80)	0.75 (-0.41 – 1.95)
Pandemic x ECERS			-0.33 (-2.02 – 1.39)		
Depression		0.44 (-0.65 – 1.54)	0.41 (-0.65 – 1.49)	1.71 (0.38 – 3.03)	0.44 (-0.64 – 1.53)
Pandemic x Depression				-3.24 (-5.17 – -1.30)	
Girl		2.39 (1.27 – 3.49)	2.34 (1.31 – 3.42)	2.41 (1.37 – 3.47)	2.61 (1.39 – 3.86)
Pandemic x Girl					-0.66 (-2.21 – 0.83)
Low birth weight		-0.76 (-2.54 – 1.06)	-0.78 (-2.51 – 0.95)	-0.69 (-2.39 – 1.03)	-0.76 (-2.48 – 0.96)
Single parent		0.43 (-5.19 – 6.07)	0.56 (-5.34 – 6.33)	0.04 (-5.86 – 5.81)	0.47 (-5.41 – 6.23)
Sibling		1.49 (0.30 – 2.66)	1.51 (0.32 – 2.68)	1.39 (0.20 – 2.56)	1.51 (0.31 – 2.68)
Working mother		-3.28 (-6.06 – -0.31)	-3.20 (-6.06 – -0.32)	-3.49 (-6.32 – -0.65)	-3.30 (-6.15 – -0.42)
Working father		1.92 (-3.57 – 7.37)	2.02 (-3.63 – 7.75)	1.38 (-4.24 – 7.09)	1.97 (-3.69 – 7.67)
Low income		-1.30 (-2.94 – 0.29)	-1.33 (-2.88 – 0.29)	-1.23 (-2.76 – 0.38)	-1.33 (-2.88 – 0.28)
Middle income		-0.36 (-1.50 – 0.79)	-0.40 (-1.55 – 0.73)	-0.31 (-1.45 – 0.82)	-0.39 (-1.54 – 0.74)
Days at nursery		0.48 (-0.91 – 1.89)	0.47 (-0.91 – 1.92)	0.42 (-0.95 – 1.85)	0.47 (-0.91 – 1.91)
Random effects					
Level 3: Nursery					
Var(Pandemic)	19.13 (7.80 – 41.13)	19.70 (8.33 – 41.42)	19.53 (8.27 – 42.50)	20.10 (8.65 – 43.36)	64.32 (28.20 – 134.81)
Var(Constant)	12.08 (4.21 – 28.31)	11.78 (4.26 – 26.08)	11.73 (4.05 – 26.72)	11.62 (3.99 – 26.87)	19.44 (8.27 – 42.05)
Level 2: Child					
Var(Pandemic)	2.12 (0.00 – 8.08)	1.80 (0.01 – 8.03)	0.42 (0.00 – 4.25)	0.36 (0.00 – 3.88)	11.63 (3.99 – 26.82)
Var(Constant)	20.58 (15.97 – 25.79)	18.82 (14.12 – 23.84)	18.91 (14.12 – 24.22)	18.65 (13.91 – 23.89)	0.43 (0.00 – 4.30)
Level 1: Time					
Var(Constant)	26.00 (22.22 – 30.13)	25.81 (21.77 – 30.02)	26.25 (22.69 – 30.39)	25.93 (22.42 – 29.96)	18.92 (14.11 – 24.19)

ECERS = the Early Childhood Environment Rating Scale, CI = credible interval. All models were also adjusted for child’s age and a propensity score.

© 2023 Sato K et al. *JAMA Pediatrics.*

eTable 22. Association Between the Pandemic and Social Relationships With Adults for 3 to 5 Years Old

Variables	Model 1<br>Coef. (95% CI)	Model 2<br>Coef. (95% CI)	Model 3<br>Coef. (95% CI)	Model 4<br>Coef. (95% CI)	Model 5<br>Coef. (95% CI)
Pandemic	-5.20 (-10.74 – 0.26)	-6.41 (-11.87 – -1.00)	-6.98 (-12.37 – -1.93)	-5.39 (-10.88 – -0.05)	-6.03 (-11.58 – -0.67)
ECERS		1.99 (0.24 – 3.74)	1.95 (-0.02 – 3.94)	1.96 (0.17 – 3.84)	2.13 (0.33 – 3.99)
Pandemic x ECERS			0.87 (-2.59 – 4.50)		
Depression		-0.61 (-2.51 – 1.33)	-0.58 (-2.51 – 1.32)	0.93 (-1.32 – 3.24)	-0.57 (-2.48 – 1.35)
Pandemic x Depression				-4.83 (-8.70 – -0.98)	
Girl		2.31 (0.61 – 4.04)	2.25 (0.63 – 3.93)	2.34 (0.72 – 4.03)	2.85 (0.90 – 4.81)
Pandemic x Girl					-1.86 (-5.10 – 1.22)
Low birth weight		-1.69 (-4.62 – 1.36)	-1.71 (-4.62 – 1.18)	-1.59 (-4.49 – 1.30)	-1.70 (-4.59 – 1.20)
Single parent		2.82 (-7.70 – 13.23)	3.08 (-7.69 – 13.84)	2.35 (-8.40 – 13.09)	2.82 (-7.91 – 13.54)
Sibling		1.30 (-0.64 – 3.16)	1.32 (-0.62 – 3.25)	1.20 (-0.76 – 3.13)	1.32 (-0.62 – 3.25)
Working mother		-2.03 (-7.02 – 3.09)	-2.05 (-7.08 – 2.93)	-2.37 (-7.34 – 2.60)	-2.09 (-7.11 – 2.93)
Working father		1.84 (-8.35 – 12.04)	2.14 (-8.22 – 12.79)	1.29 (-9.07 – 11.86)	1.87 (-8.45 – 12.46)
Low income		-2.26 (-4.96 – 0.46)	-2.31 (-4.89 – 0.46)	-2.18 (-4.76 – 0.60)	-2.30 (-4.89 – 0.48)
Middle income		-1.20 (-3.04 – 0.72)	-1.25 (-3.14 – 0.62)	-1.14 (-3.03 – 0.75)	-1.23 (-3.11 – 0.66)
Days at nursery		0.12 (-2.28 – 2.51)	0.11 (-2.31 – 2.56)	0.02 (-2.40 – 2.47)	0.10 (-2.32 – 2.56)
Random effects					
Level 3: Nursery					
Var(Pandemic)	90.73 (38.86 – 193.95)	89.93 (38.48 – 191.15)	87.73 (36.82 – 193.85)	90.77 (38.56 – 198.54)	64.32 (28.20 – 134.81)
Var(Constant)	14.09 (4.17 – 34.35)	17.16 (5.29 – 41.28)	17.56 (5.32 – 40.92)	17.00 (5.06 – 40.38)	89.54 (38.04 – 197.69)
Level 2: Child					
Var(Pandemic)	45.81 (13.74 – 77.10)	49.35 (21.43 – 82.00)	48.26 (20.43 – 78.71)	46.48 (19.32 – 76.59)	17.36 (5.35 – 40.94)
Var(Constant)	23.33 (9.70 – 37.79)	20.41 (4.35 – 33.94)	20.89 (4.93 – 35.43)	21.11 (5.42 – 35.39)	47.58 (20.26 – 78.15)
Level 1: Time					
Var(Constant)	97.46 (80.46 – 115.66)	97.22 (80.05 – 116.40)	96.88 (80.32 – 118.41)	96.32 (80.12 – 117.90)	20.84 (4.97 – 35.31)

ECERS = the Early Childhood Environment Rating Scale, CI = credible interval. All models were also adjusted for child’s age and a propensity score.

© 2023 Sato K et al. *JAMA Pediatrics.*

eTable 23. Association Between the Pandemic and Discipline for 3 to 5 Years Old

Variables	Model 1<br>Coef. (95% CI)	Model 2<br>Coef. (95% CI)	Model 3<br>Coef. (95% CI)	Model 4<br>Coef. (95% CI)	Model 5<br>Coef. (95% CI)
Pandemic	-5.01 (-9.60 – -0.40)	-5.69 (-10.24 – -1.21)	-6.13 (-10.62 – -1.83)	-5.02 (-9.57 – -0.64)	-5.41 (-10.06 – -0.88)
ECERS		1.67 (0.29 – 3.06)	1.83 (0.30 – 3.34)	1.65 (0.26 – 3.06)	1.77 (0.38 – 3.18)
Pandemic x ECERS			-0.42 (-3.58 – 2.81)		
Depression		-0.17 (-1.67 – 1.37)	-0.17 (-1.64 – 1.34)	0.83 (-0.95 – 2.61)	-0.14 (-1.68 – 1.36)
Pandemic x Depression				-3.72 (-7.11 – -0.44)	
Girl		2.12 (0.87 – 3.40)	2.10 (0.83 – 3.35)	2.15 (0.89 – 3.40)	2.51 (1.03 – 3.97)
Pandemic x Girl					-1.52 (-4.43 – 1.20)
Low birth weight		-1.18 (-3.45 – 1.12)	-1.23 (-3.51 – 1.05)	-1.13 (-3.41 – 1.15)	-1.20 (-3.48 – 1.08)
Single parent		-0.18 (-8.85 – 8.29)	-0.10 (-8.83 – 8.80)	-0.51 (-9.22 – 8.41)	-0.20 (-8.90 – 8.72)
Sibling		1.78 (0.28 – 3.22)	1.78 (0.29 – 3.27)	1.71 (0.22 – 3.19)	1.79 (0.29 – 3.26)
Working mother		-0.25 (-4.17 – 3.77)	-0.18 (-4.10 – 3.76)	-0.50 (-4.35 – 3.41)	-0.27 (-4.14 – 3.66)
Working father		1.60 (-6.88 – 10.10)	1.69 (-6.65 – 10.36)	1.26 (-7.04 – 9.93)	1.61 (-6.68 – 10.29)
Low income		0.64 (-1.43 – 2.79)	0.58 (-1.45 – 2.69)	0.67 (-1.35 – 2.80)	0.61 (-1.41 – 2.74)
Middle income		0.13 (-1.33 – 1.60)	0.11 (-1.36 – 1.62)	0.18 (-1.29 – 1.67)	0.13 (-1.33 – 1.62)
Days at nursery		1.71 (-0.20 – 3.57)	1.70 (-0.17 – 3.60)	1.63 (-0.23 – 3.54)	1.70 (-0.17 – 3.60)
Random effects					
Level 3: Nursery					
Var(Pandemic)	66.12 (28.14 – 141.00)	64.32 (28.20 – 134.81)	64.30 (26.93 – 138.74)	63.64 (27.48 – 136.23)	64.32 (28.20 – 134.81)
Var(Constant)	20.90 (8.19 – 45.83)	19.22 (7.73 – 42.63)	19.47 (7.56 – 42.70)	19.21 (7.35 – 42.78)	62.65 (26.85 – 134.16)
Level 2: Child					
Var(Pandemic)	54.00 (33.15 – 76.67)	58.93 (39.58 – 82.12)	58.44 (37.73 – 81.28)	57.32 (37.10 – 79.72)	19.16 (7.36 – 42.85)
Var(Constant)	3.33 (0.01 – 10.89)	1.36 (0.01 – 6.60)	1.19 (0.00 – 5.42)	1.21 (0.00 – 5.44)	58.36 (37.80 – 81.05)
Level 1: Time					
Var(Constant)	69.85 (59.54 – 81.06)	68.95 (59.20 – 78.78)	69.05 (59.75 – 78.85)	68.86 (59.64 – 78.56)	1.22 (0.00 – 5.46)

ECERS = the Early Childhood Environment Rating Scale, CI = credible interval. All models were also adjusted for child's age and a propensity score.

© 2023 Sato K et al. *JAMA Pediatrics.*

eFigure 5. Marginal Association Between Parental Depression and Child Development

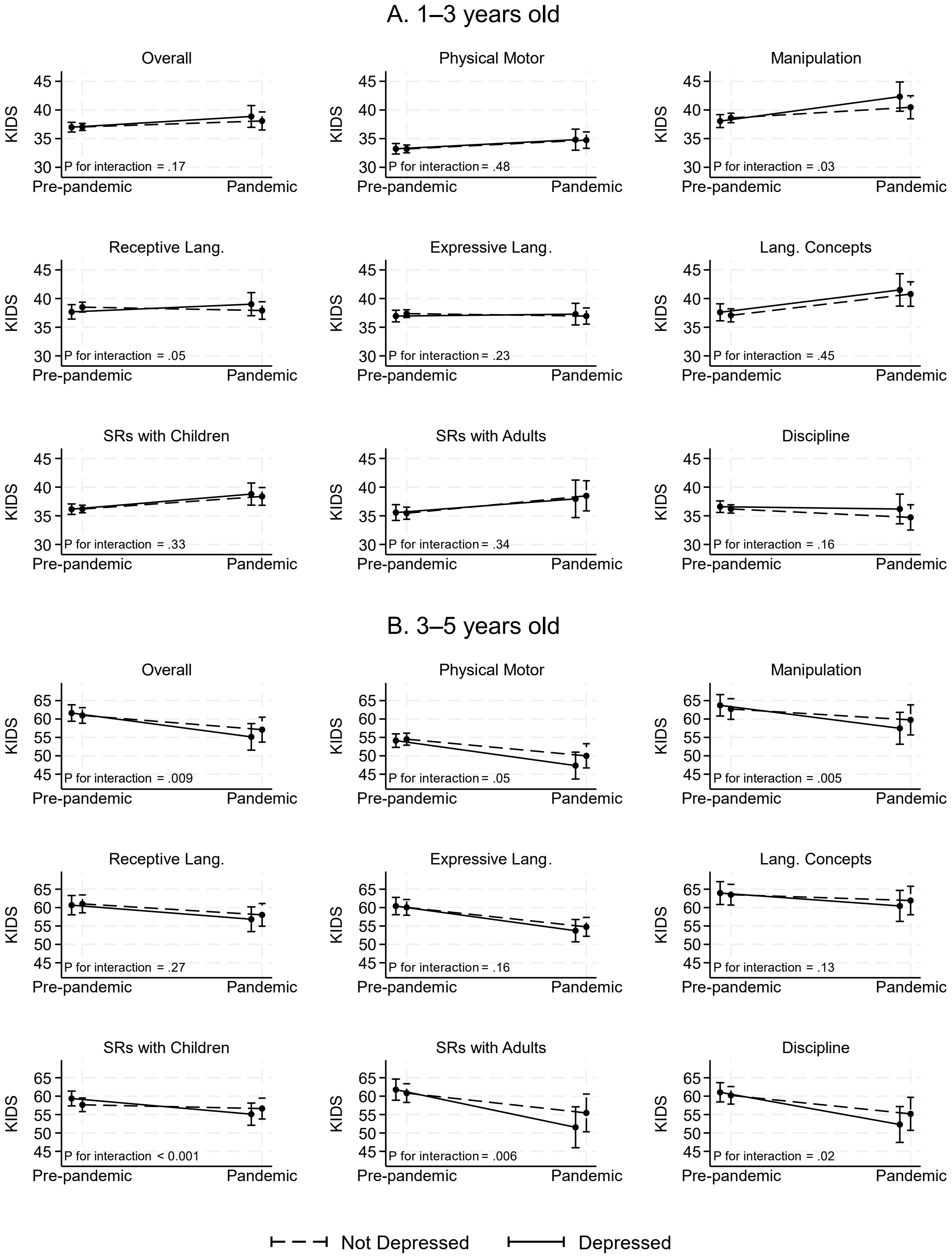

Lang. = Language, SR = social relationship. All models were adjusted for the quality of care, parental depression, child's sex, low birth weight, single parent, sibling, working status of mother and father, household income, number of days in the nursery, child's age, and a propensity score.

© 2023 Sato K et al. *JAMA Pediatrics.*

eFigure 6. Marginal Association Between Sex and Child Development

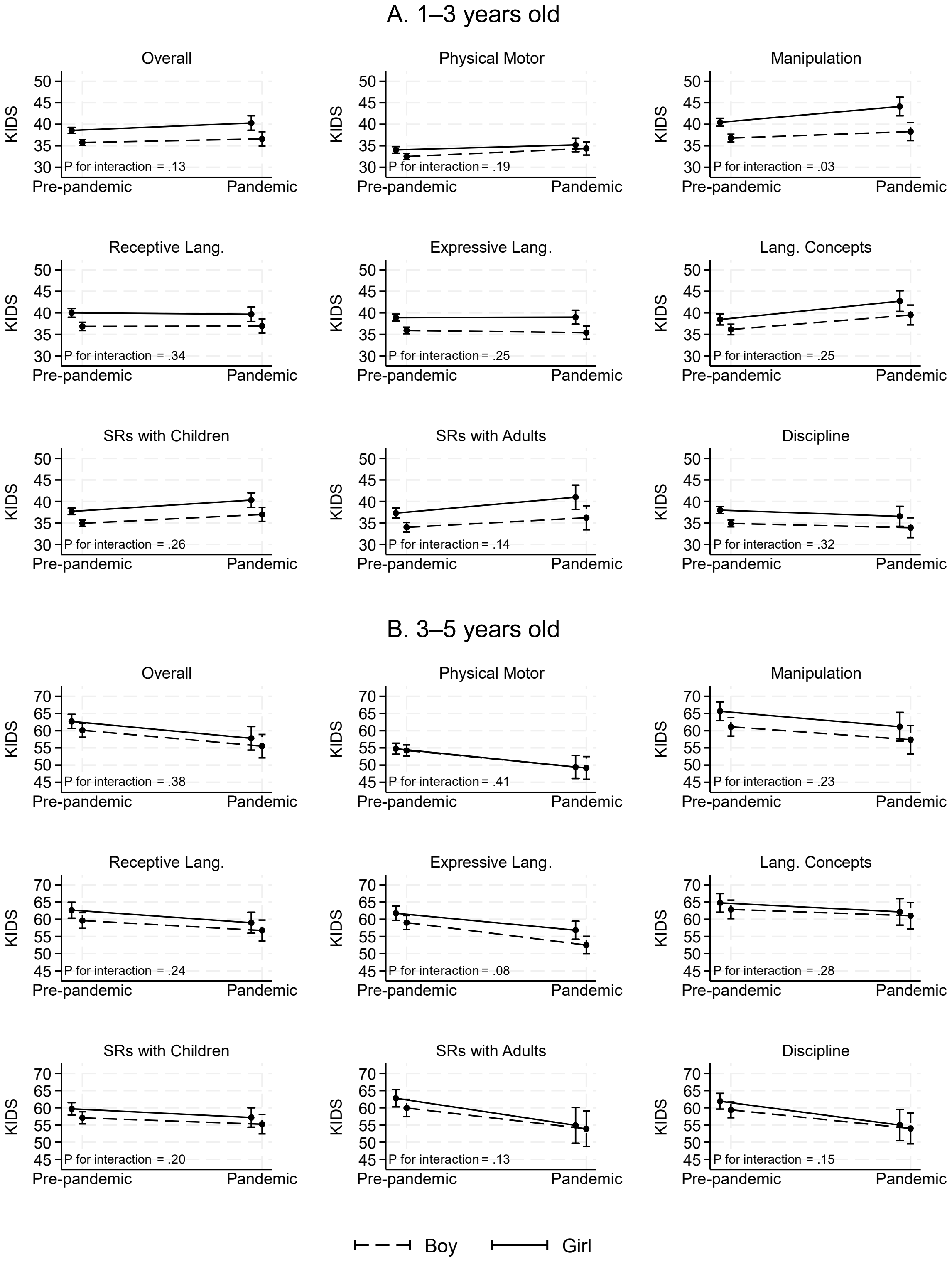

Lang. = Language, SR = social relationship. All models were adjusted for the quality of care, parental depression, child's sex, low birth weight, single parent, sibling, working status of mother and father, household income, number of days in the nursery, child's age, and a propensity score.

© 2023 Sato K et al. *JAMA Pediatrics.*

eFigure 7. Association Between the Pandemic and Child Development Without Adjustment of Propensity Scores

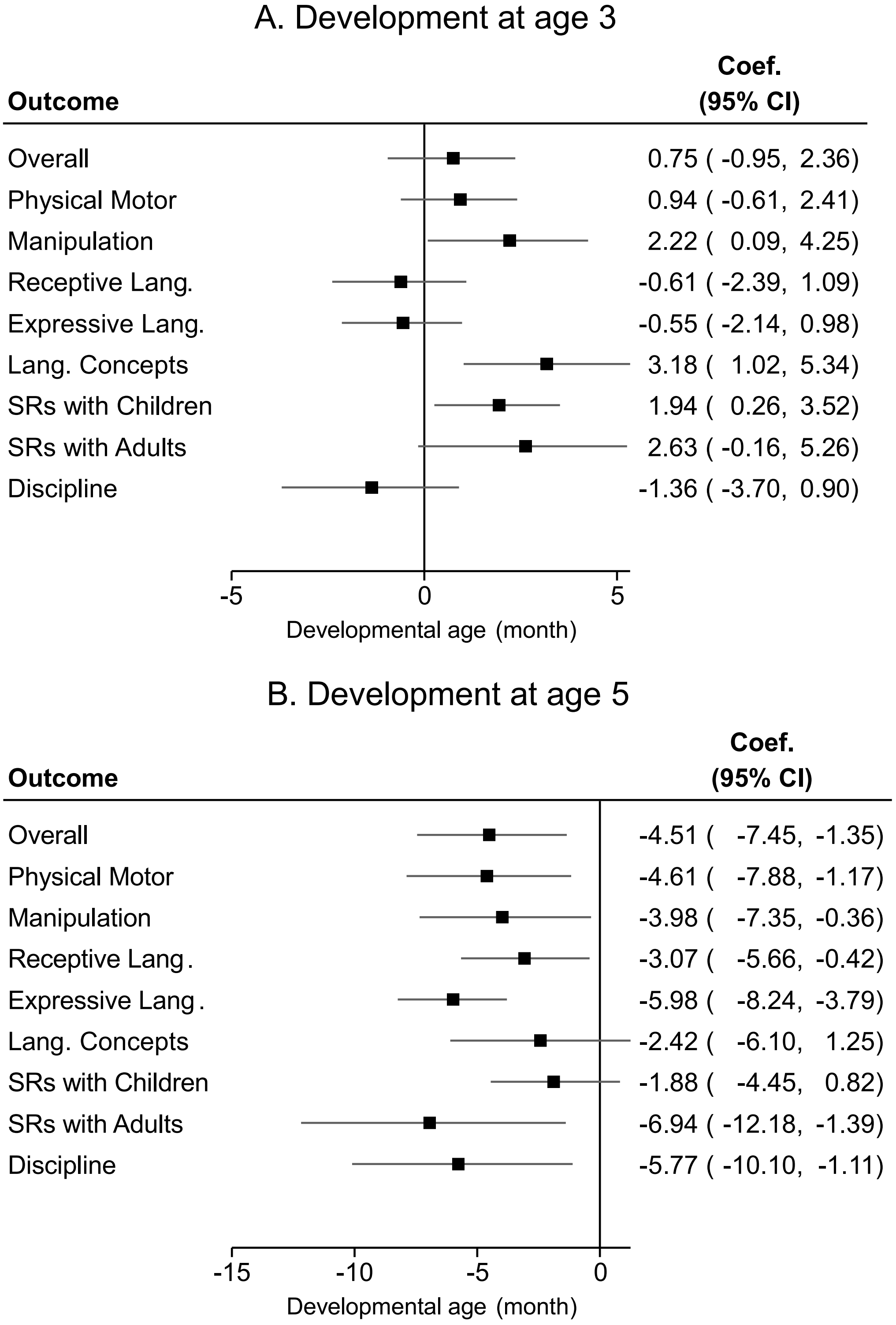

Coef. = coefficient, CI = credible interval, Lang. = Language, SR = social relationship. All models were adjusted for the quality of care, parental depression, child's sex, low birth weight, single parent, sibling, working status of mother and father, household income, number of days in the nursery, and child's age.

© 2023 Sato K et al. *JAMA Pediatrics.*

eFigure 8. Association between the Pandemic and Child Development Nested Within the Nursery at Follow-up

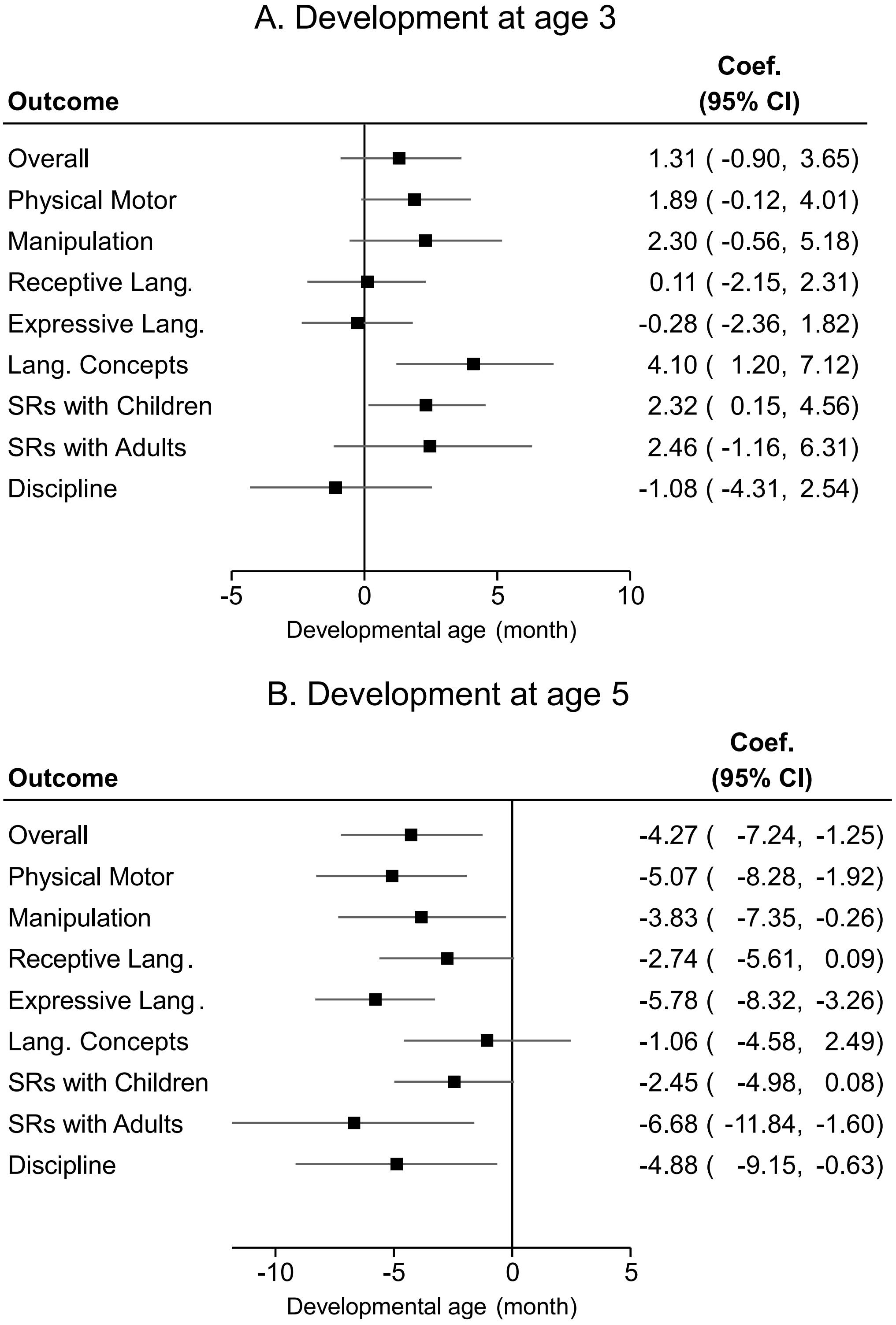

Coef. = coefficient, CI = credible interval, Lang. = Language, SR = social relationship. All models were adjusted for the quality of care, parental depression, child's sex, low birth weight, single parent, sibling, working status of mother and father, household income, number of days in the nursery, child's age, and a propensity score.

© 2023 Sato K et al. *JAMA Pediatrics.*

eTable 24. Number of Missing Values

	Cohort aged 1 to 3 (n = 894)	Cohort aged 3 to 5 (n = 880)
Low birth weight	10 (1.1)	5 (0.6)
Single parent	1 (0.1)	1 (0.1)
Having a sibling	3 (0.3)	1 (0.1)
Working mother	13 (1.5)	9 (1.0)
Working father	14 (1.6)	11 (1.3)
Household income	9 (1.0)	7 (0.8)
Days in nursery	7 (0.8)	4 (0.5)
Depression	27 (3.0)	20 (2.3)
KIDS		
Overall	94 (10.5)	87 (9.9)
Physical Motor	30 (3.4)	26 (3.0)
Manipulation	26 (2.9)	19 (2.2)
Receptive Lang.	24 (2.7)	22 (2.5)
Expressive Lang.	21 (2.3)	26 (3.0)
Lang. Concepts	13 (1.5)	15 (1.7)
SRs with Children	17 (1.9)	19 (2.2)
SRs with Adults	21 (2.3)	18 (2.0)
Discipline	25 (2.8)	18 (2.0)

KIDS = the Kinder Infant Development Scale, Lang. = Language, SR = social relationship. % of missing values is in parenthesis.

eFigure 9. Complete Case Analysis for the Association Between the Pandemic and Child Development

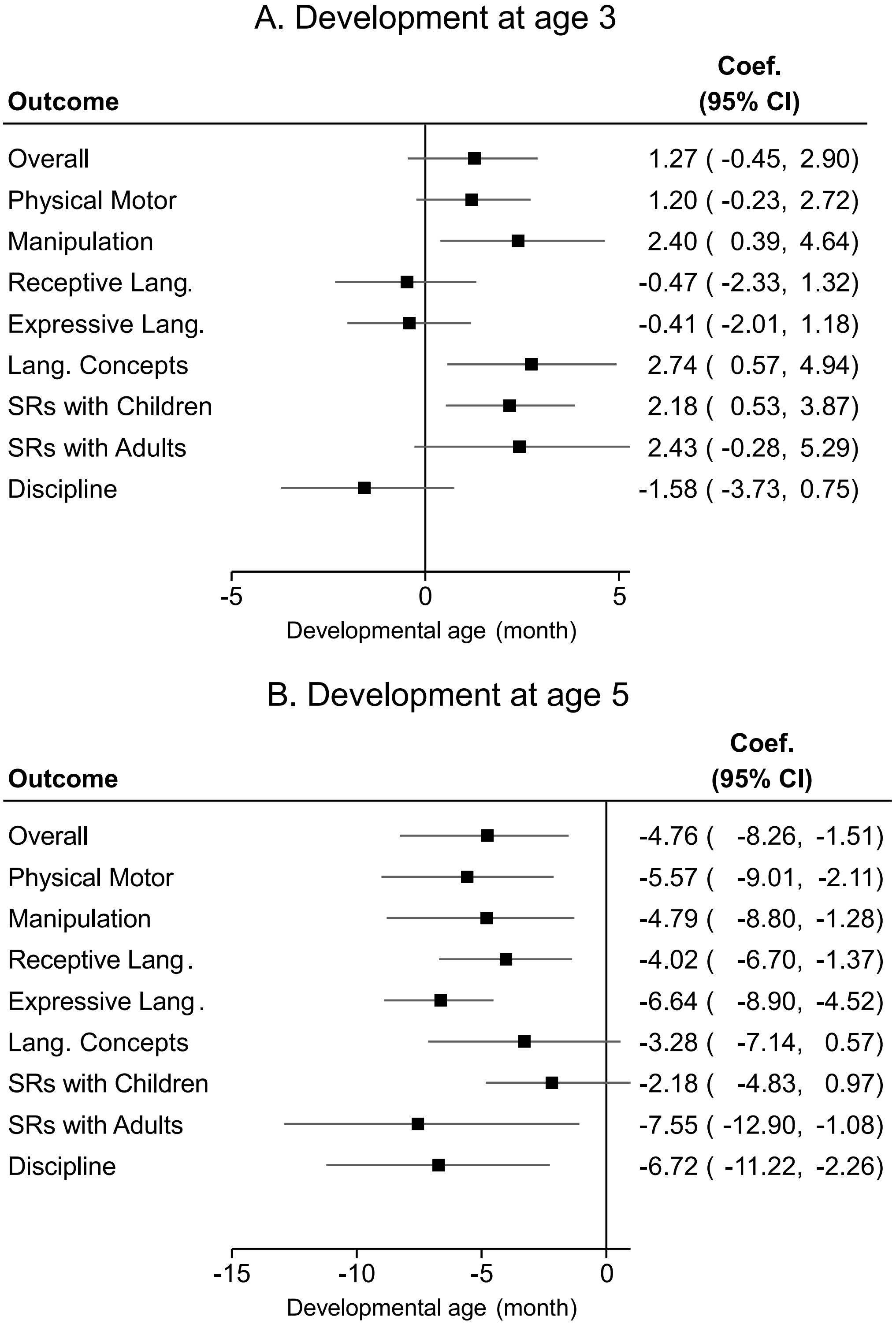

Coef. = coefficient, CI = credible interval, Lang. = Language, SR = social relationship. All models were adjusted for the quality of care, parental depression, child's sex, low birth weight, single parent, sibling, working status of mother and father, household income, number of days in the nursery, child's age, and a propensity score.

© 2023 Sato K et al. *JAMA Pediatrics.*

eFigure 10. Subgroup Analysis by Follow-up Timing for the Association Between the Pandemic and Child Development

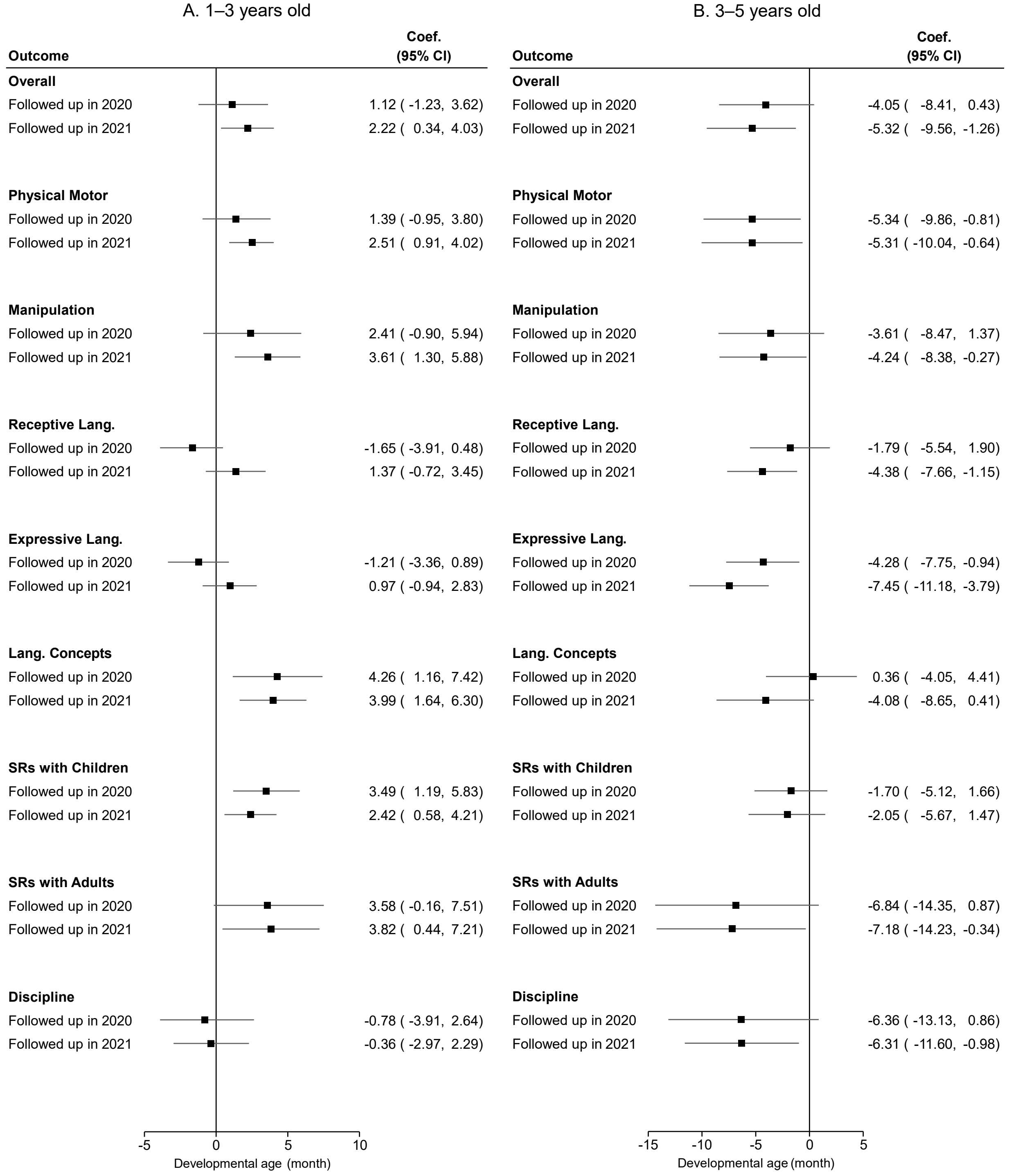

Coef. = coefficient, CI = credible interval, Lang. = Language, SR = social relationship. All models were adjusted for the quality of care, parental depression, child's sex, low birth weight, single parent, sibling, working status of mother and father, household income, number of days in the nursery, child's age, and a propensity score.

© 2023 Sato K et al. *JAMA Pediatrics.*

eFigure 11. Association Between the Pandemic and Child Development Using a Saturated Function for Age

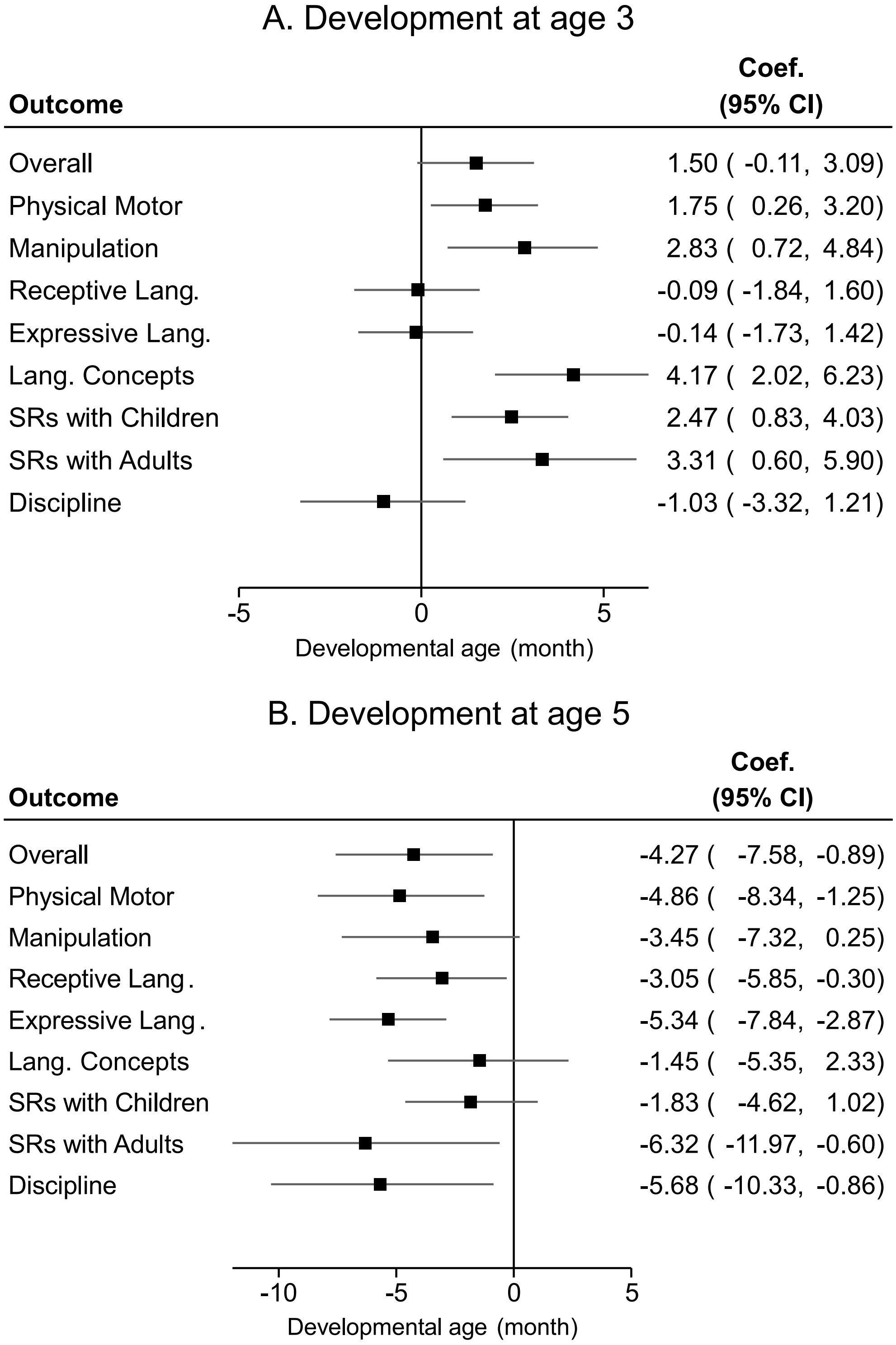

Coef. = coefficient, CI = credible interval, Lang. = Language, SR = social relationship. All models were adjusted for the quality of care, parental depression, child's sex, low birth weight, single parent, sibling, working status of mother and father, household income, number of days in the nursery, and dummy variables for each month of age.

© 2023 Sato K et al. *JAMA Pediatrics.*
