# Supplementary material for: Association Between the COVID-19 Pandemic and Early Childhood Development
Source: JAMA Pediatr. 2023 Jul 10;177(9):930–8. doi: 10.1001/jamapediatrics.2023.2096 (PMC10334298; doi:10.1001/jamapediatrics.2023.2096)
Supplement: Supplement 2. — Data Sharing Statement [file jamapediatr-e232096-s002.pdf]

Data Sharing Statement

Sato. Association Between the COVID-19 Pandemic and Early Childhood Development. *JAMA Pediatr*. Published July 10, 2023. doi:10.1001/jamapediatrics.2023.2096

Data
Data available: No

Additional Information
Explanation for why data not available: The data are not publicly available due to participants' privacy and consent. The data that support the findings of this study are available on request to the corresponding author.
